# Examining the evidence for mutual modulation between m6A modification and circular RNAs: current knowledge and future prospects

**DOI:** 10.1186/s13046-024-03136-2

**Published:** 2024-08-03

**Authors:** Xiaozhu Tang, Mengjie Guo, Yuanjiao Zhang, Junxian Lv, Chunyan Gu, Ye Yang

**Affiliations:** 1 Nanjing Hospital of Chinese Medicine Affiliated to Nanjing University of Chinese Medicine, Nanjing, China; 2 School of Medicine, Nanjing University of Chinese Medicine, Nanjing, China

**Keywords:** Circular RNAs, N6-methyladenosine, Crosstalk, Cancer, Therapeutic resistance

## Abstract

The resistance of cancer cells to treatment significantly impedes the success of therapy, leading to the recurrence of various types of cancers. Understanding the specific mechanisms of therapy resistance may offer novel approaches for alleviating drug resistance in cancer. Recent research has shown a reciprocal relationship between circular RNAs (circRNAs) and N6-methyladenosine (m6A) modification, and their interaction can affect the resistance and sensitivity of cancer therapy. This review aims to summarize the latest developments in the m6A modification of circRNAs and their importance in regulating therapy resistance in cancer. Furthermore, we explore their mutual interaction and exact mechanisms and provide insights into potential future approaches for reversing cancer resistance.

## Introduction

Up to now, more than 160 varieties of chemical alterations have been identified in RNA molecules, with methylation being the most prevalent form [1]. These RNA methylation modifications include N6-methyladenosine (m6A), 5-methylcytosine (m5C), 7-methylguanine (m7G), 2'-O-dimethyladenosine (m6Am), N1-methyladenosine (m1A), and 5-hydroxymethylcytosine (5hmC) [2]. Among these modifications, m6A is the most prevalent modification in eukaryotic cells [3]. Also known as N6-methyladenosine modification, m6A is a type of post-transcriptional RNA modification that plays a crucial role in regulating gene expression [4, 5]. The discovery of m6A modification in RNA was first reported in the 1970s [6], and it has essential functions in various processes, such as RNA stability, splicing, translation, and RNA–protein interactions [7].

Circular RNAs (circRNAs) are a type of RNA molecule that forms a single-stranded closed loop structure, lacking the typical 5' to 3' ends [8]. Researchers now widely accept that circRNAs can be translated into functional peptides through internal ribosome entry site (IRES)-mediated translation. The absence of a 5' cap in circRNAs suggests that they must rely on cap-independent mechanisms, such as m6A modification, for translation initiation. Unlike linear RNA molecules, circRNAs resist degradation by exonucleases and exhibit more excellent stability [9]. The topic of m6A modification in circRNAs is an area of ongoing research and still needs to be fully understood. Initially, it was believed that circRNAs, due to their circular structure, were not susceptible to m6A modification [10]. However, recent studies have challenged this notion by providing evidence of m6A modifications in circRNAs.

**Fig. 1:**
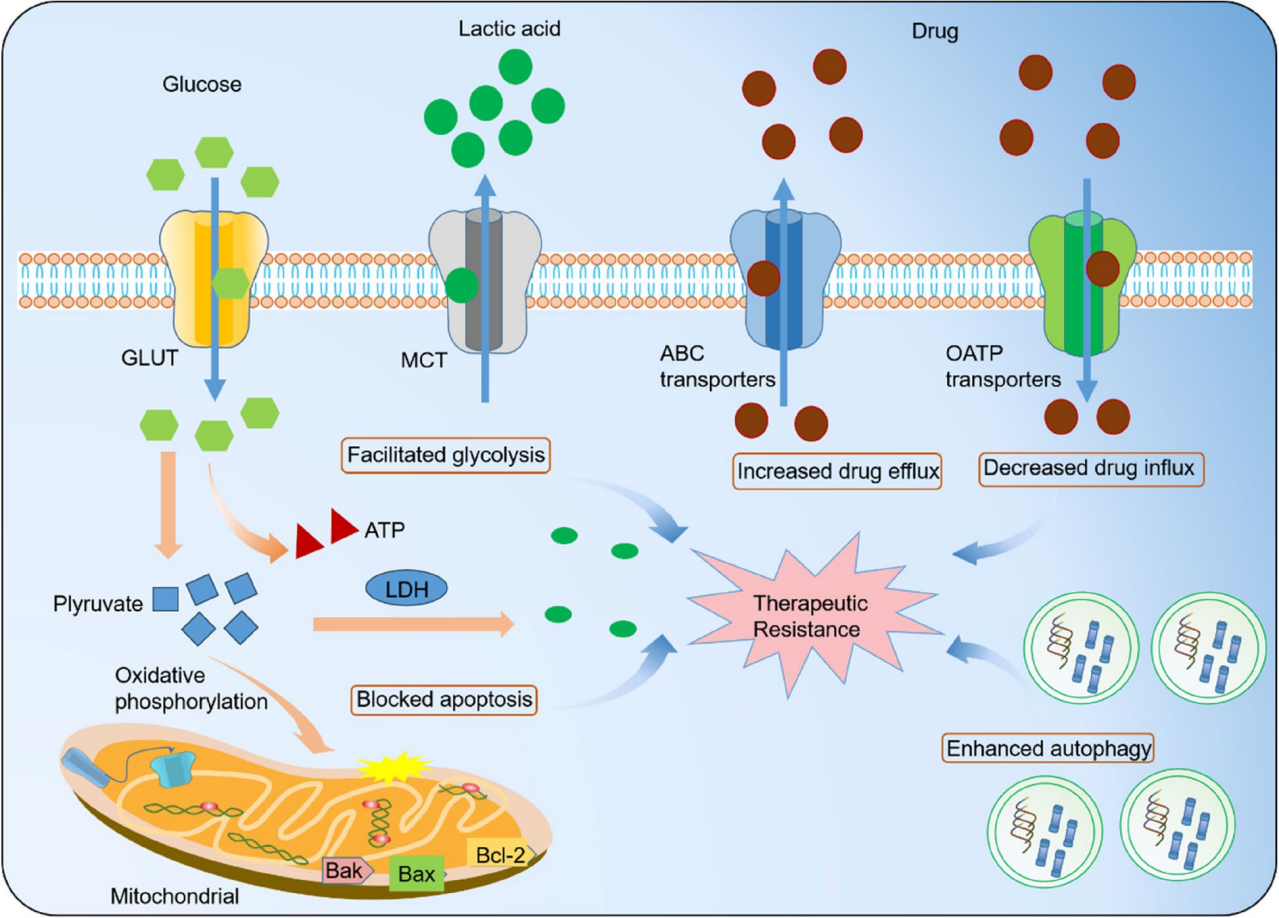
Mechanisms of therapeutic resistance in cancer. Enhanced drug efflux and diminished drug influx result in a reduced accumulation of drugs within cancer cells. In addition, inhibiting programmed cell death, facilitating DNA damage repair, and augmenting cellular self-digestion processes also contribute to the survival of tumor cells under treatment-induced stress. Furthermore, cancer cells prefer glucose metabolism, which produces lactic acid through glycolysis, promoting rapid cell proliferation in response to therapeutic interventions. Lastly, an imbalanced tumor microenvironment (TME), promotion of the transition from epithelial to EMT, and heightened properties of cancer stem cells (CSCs) also impede the effectiveness of cancer treatment

A growing body of experimental data has elucidated that m6A modification can also occur in circRNAs [11]. This modification can potentially influence the stability, localization, and function of circRNAs [12]. It has been suggested that m6A modification in circRNAs can regulate their interactions with RNA-binding proteins (RBPs), miRNAs, and specific molecules, thereby impacting their overall cellular functions [13]. However, our understanding of m6A modification in circRNAs is still relatively limited, and further studies are urgently needed to investigate the underlying mechanisms and functional significance [14]. Nevertheless, the study of m6A modification in circRNAs has great potential for uncovering novel regulatory mechanisms in RNA biology [15]. Under treatment pressure, cancer cells can develop resistance to therapy, allowing them to evade death. On one hand, changes in drug transport and metabolism can impair the efficacy of various anticancer drugs. On the other hand, cancer cells can acquire survival advantages through mechanisms like apoptosis resistance, DNA damage repair, and induction of epithelial-to-mesenchymal transition (EMT) (Fig. 1).

Herein, this review comprehensively analyzes the latest developments and mechanisms of m6A modification of circRNA in tumor treatment, the crosstalk between m6A modifications and circRNAs, and their interactions in modulating treatment resistance. This highlights the potential of targeting these modifications and RNAs to overcome resistance to cancer treatment.

## m6A modification and circRNAs

### m6A modification regulators

**Fig. 2:**
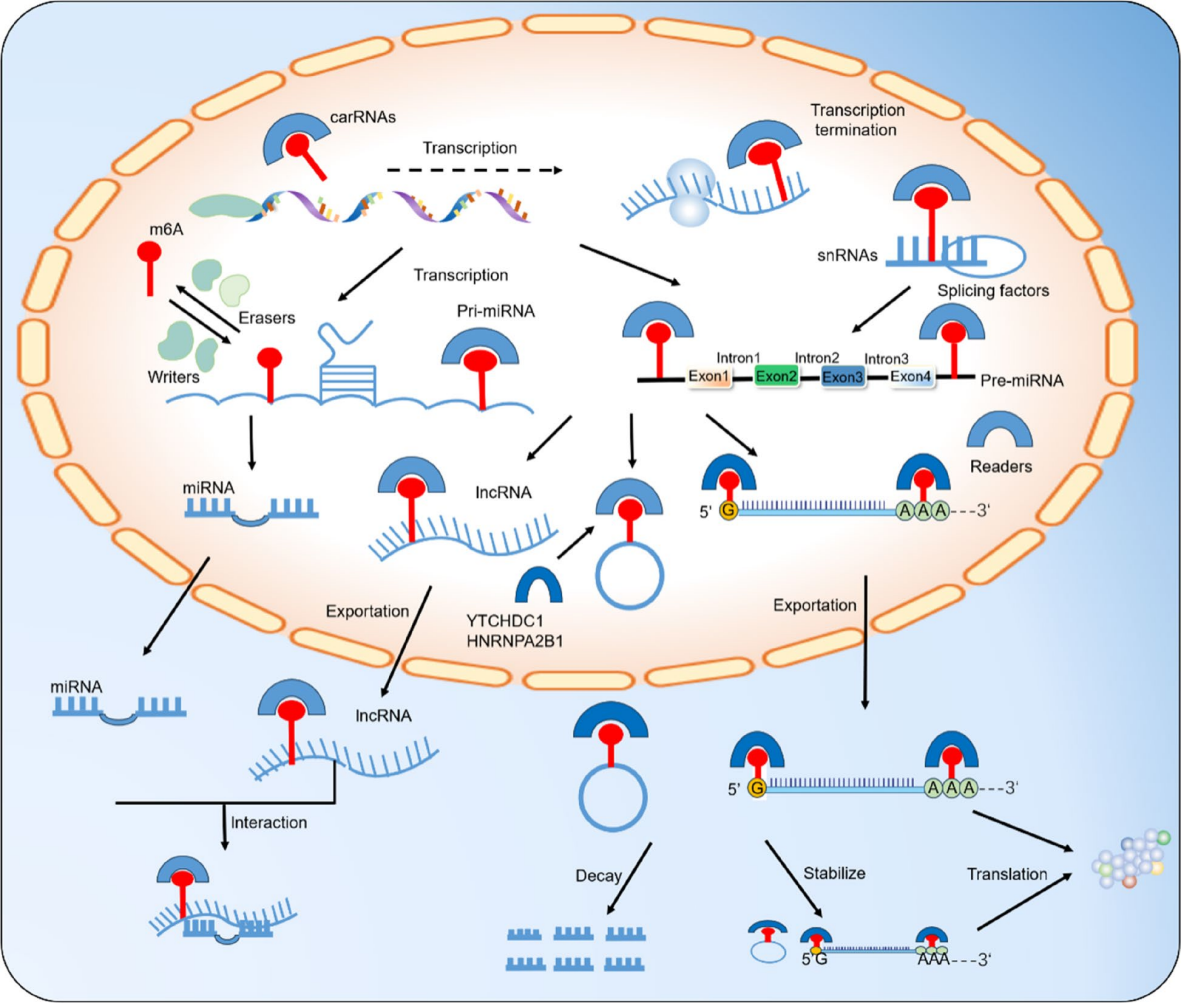
Overview of m6A modification. A complex of multicomponent m6A methyltransferases installs the writers of the m6A modification, while the erasers remove it through demethylases. In the nucleus, nuclear readers recognize m6A and regulate RNA transcription, splicing, and structure. In the cytoplasm, cytoplasmic readers detect m6A and regulate RNA stability, translation, and binding capacity

Readers are crucial in various aspects of RNA processing, including RNA splicing, transport, degradation, and translation [16]. Some well-known reader proteins include the YTH domain-containing family (YTHDF) proteins and YTH domain-containing family (YTHDC) proteins [17]. The interaction among writers, erasers, and readers of m6A modification regulates RNA processing and function [18]. The addition and removal of the methyl group can affect RNA stability, splicing, translation efficiency, and RNA–protein interactions [10]. The reader proteins determine the fate of the modified RNA, directing it toward degradation or translation [19]. A concise overview is depicted in Fig. 2.

The YTH domain functions as a reader molecule, specifically recognizing m6A modifications on RNA in a methylation-dependent manner. Humans have five known YTH domain-containing proteins: YTHDC1, YTHDC2, YTHDF1, YTHDF2, and YTHDF3. These proteins play a critical role in post-transcriptional regulation, influencing splicing, translation, RNA localization, and overall RNA lifespan [20]. A comparison of the crystal structures of YTHDC2 and YTHDC1 domains reveals that both a conserved hydrophobic pocket and a positively charged surface are crucial for recognizing m6A-modified RNA [21]. Similarly, YTHDF1, YTHDF2, and YTHDF3 also possess aromatic cages, specific residues for m6A recognition, and a basic patch that facilitates RNA backbone binding [22]. Scutenaire et al. conducted a comprehensive evolutionary analysis of YTH domains in Viridiplantae [23]. Their study revealed that vascular plants possess YTHDF- and YTHDC-type motifs, which contain the essential amino acids for RNA binding and m6A accommodation. These motifs are predicted to adopt a similar structural fold as YTH domains found in animals and yeast.

Within the cell nucleus, the dynamic regulation of RNA methylation is carried out by m6A writers and erasers. For instance, YTHDC1 plays a crucial role in splicing and controlling the export of m6A-modified mRNAs. This is achieved by recruiting SRSF3 while simultaneously blocking the binding of SRSF10 to the mRNA. YTHDF1 recognizes m6A-containing mRNAs and promotes both initiation and elongation of translation in the cytoplasm. Additionally, YTHDF2 recognizes the same mRNAs and targets them for degradation through deadenylation mediated by the CCR4-NOT complex and endoribonucleolytic cleavage mediated by HRSP12. Notably, YTHDF3 interacts with YTHDF1 and YTHDF2, accelerating the overall metabolism of m6A-modified mRNAs. YTHDC2, on the other hand, plays a distinct role in regulating the switch from mitosis to meiosis through its interaction with MEIOC. Additionally, YTHDC2 destabilizes target RNAs by interacting with proteins such as XRN1, UPF1, and MOV10. Interestingly, YTHDC2 also binds to the 18S ribosomal RNA and exhibits 3'-5' RNA helicase activity, both of which contribute to the translation of its target RNAs.

### Writers, erasers, and readers actively interact in the process of m6A modification

The m6A modification process is controlled by a trio of proteins known as writers, erasers, and readers. These proteins, including METTL3/14/16, VIRMA, and WTAP, add a methyl group to the RNA, effectively turning the switch on. Conversely, erasers, such as FTO and ALKBH proteins, remove the methyl group, acting as molecular "off switches". Additionally, reader proteins like YTHDC1/2 and IGF2BP1/2/3 act as cellular sensors, detecting the presence or absence of the m6A mark and triggering downstream events accordingly. This dynamic interplay allows for precise control over the fate and function of RNA.

The intricate process of RNA m6A modification is dependent on the dynamic interaction between writers, readers, and erasers within eukaryotic cells [24]. These specialized proteins, including methyltransferases (writers) such as METTL3, METTL14, and WTAP, work together to form the m6A methyltransferase complex (MTC) responsible for adding m6A to target mRNA [25, 26]. Reader proteins in both the nucleus and cytoplasm act as chemical regulators. They can directly influence RNA processing by recruiting specific partners such as YTHDC1, YTHDF2, and YTHDF3, which can alter RNA base-pairing, secondary structures, and protein-RNA interactions, ultimately determining the fate of the RNA [27].

Erasers, also known as enzymes, play a crucial role in this dynamic dance by removing the m6A modification and creating a reversible system alongside writers and readers [28, 29]. This dynamic regulation of m6A has become a critical area of cancer research, as it is closely linked to how environmental pollutants can trigger carcinogenesis.

### Mutual regulation between circRNAs and m6A modification

CircRNAs can be classified into four categories: circular intronic RNAs (ciRNAs), exonic circRNAs (ecRNAs), tRNA intronic circRNAs (tricRNAs), and exon–intron circRNAs (EIciRNAs), each with distinct formation mechanisms. Researchers have elucidated several biogenesis mechanisms, including lariat-driven circularization and circularization associated with RBPs.

**Fig. 3:**
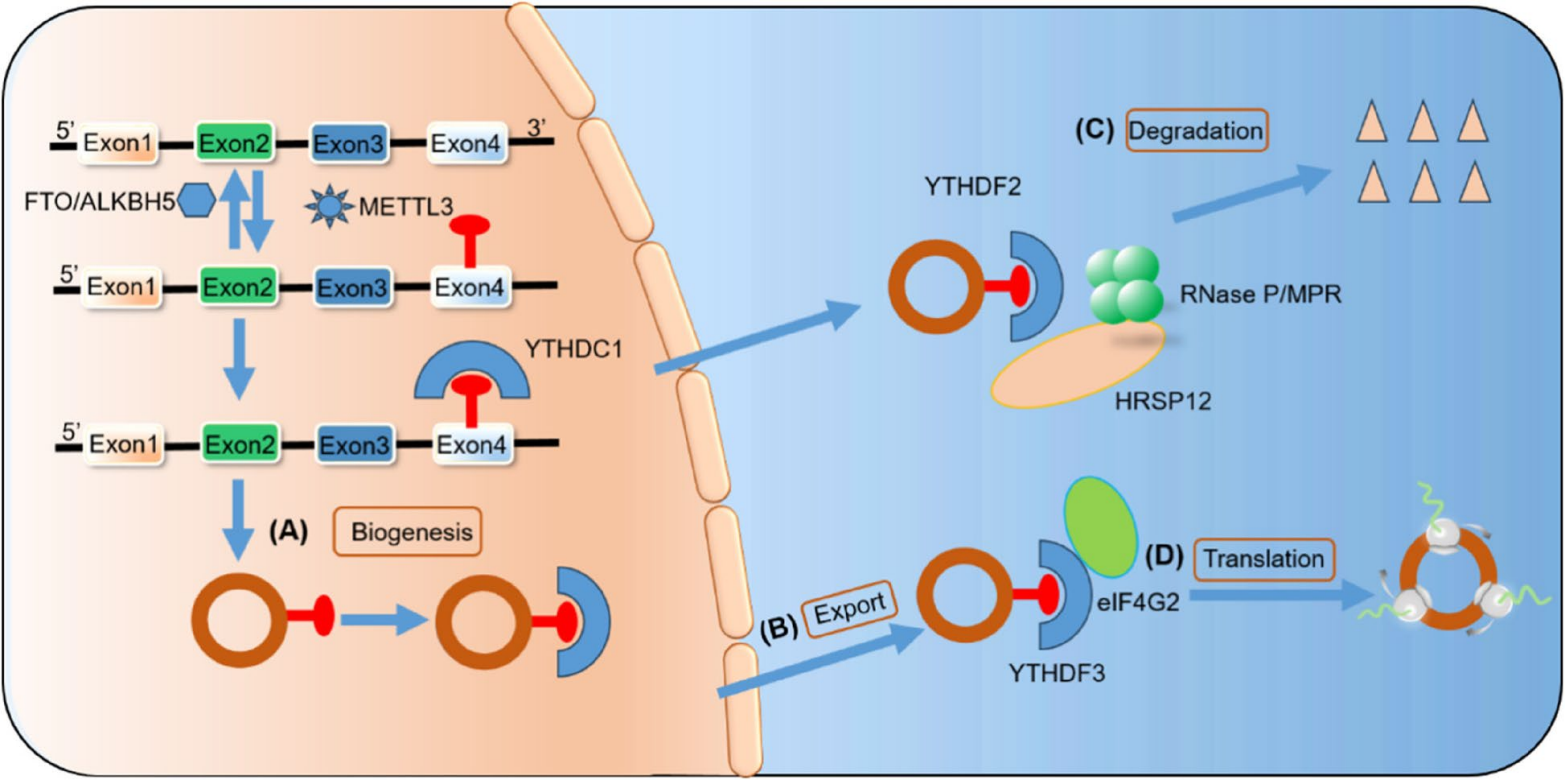
Impact of m6A modification on biosynthesis, export, translation, and degradation of circRNAs. **A** CircRNA biosynthesis: METTL3, an m6A writer, adds m6A sites to pre-mRNAs. These m6A sites can then be detected by YTHDC1, an m6A reader, which facilitates the process of back-splicing. **B** CircRNA export: YTHDC1 also promotes the export of circRNAs to the cytoplasm by binding to m6A residues. **C** CircRNA degradation: YTHDF2, another m6A reader, recognizes m6A-modified circRNAs and forms a complex with RNase P/MRP and HRSP12, leading to the degradation of circRNAs. **D** CircRNA translation: YTHDF3 and eIF4G2 play vital roles in initiating the translation of m6A-modified circRNAs

Surprisingly, emerging research has revealed that m6A fulfills an essential role in various aspects of circRNAs. These aspects are illustrated in Fig. 3 and include: (1) Biosynthesis of circRNAs. Previous studies have shown that m6A modulates the biosynthesis of circZNF609 in a YTHDC1/METTL3-dependent manner. Similarly, m6A modification is strongly related to the biogenesis of circ1662, circARL3, and circMETTL3 in various tumors. (2) Export of circRNAs from the nucleus to the cytoplasm. Exporting circRNAs from the nucleus to the cytoplasm is essential for their function. m6A-modified circNSUN2 can interact with YTHDC1 in the nucleus, thereby facilitating its export from the nucleus to the cytoplasm. (3) Degradation of circRNAs. There is limited research on the degradation of circRNAs, and the potential mechanisms are still unclear. However, m6A modification has been found to suppress the degradation of circRNAs, thereby increasing their stability. For example, a study showed that the t_1/2_ of m6A-modified circCUX1 was extended when treated with Actinomycin D, indicating that m6A modification enhances the stability of circCUX1 [30]. (4) Translation of circRNAs. CircRNAs can be translated through two cap-independent pathways: m6A-dependent translation and the IRES. The translation of m6A-modified circRNAs is dependent on eIF4G2 and the involvement of METTL3/14.

## m6A modulates circRNA subcellular localization

The m6A modification plays a crucial role in regulating the subcellular localization of RNA. This modification affects the binding of RBPs to RNA molecules, which influences their localization within the cell [31]. RBPs recognize the m6A modification on RNA and bind to the modified RNA molecules. These RBPs then have the ability to identify and transport the RNA to specific subcellular compartments, such as the nucleus or cytoplasm [32]. For example, the YTH domain-containing proteins, which are RBPs that specifically recognize m6A, can shuttle between the nucleus and cytoplasm, facilitating the transport of m6A-modified RNA [33].

CircRNAs localize to various cellular compartments, such as the nucleus or cytoplasm, interacting with other molecules to influence gene expression. However, disruptions in circRNA localization can hinder these interactions, potentially affecting protein partners and leading to cellular dysfunction or disease. Therefore, understanding the location of a circRNA is crucial in comprehending its regulatory mechanisms, particularly in the cytoplasmic competitive endogenous RNA (ceRNA) model. For example, in colorectal cancer (CRC), the m6A reader YTHDC1 promotes the export of circNSUN2 to the cytoplasm, where it forms a circNSUN2/IGF2BP2/HMGA2 RNA–protein complex that stabilizes HMGA2 mRNA. This complex has been shown to drive cancer metastasis by promoting the epithelial-to-mesenchymal transition (EMT) process [34].

A previous study found a weak correlation between the cytoplasmic localization and stability of circRNAs [35]. This suggests that long-lived circRNAs accumulate in the cytoplasm, likely during mitosis when the nucleus disassembles. The subcellular localization of circRNAs can also be affected by m6A modification, which can target them to specific cellular compartments [10]. Similarly, another study showed that m6A modification of the circRNA circDENND4C suppressed its export from the nucleus to the cytoplasm. In addition to regulating the export of circRNAs to the cytoplasm, m6A modification may also impact the localization of circRNAs within the cytoplasm [36]. For example, m6A modification of the circSRY promotes its localization to stress granules [37].

### m6A modulates the expression of circRNAs

Previous studies have demonstrated that METTL3 is responsible for installing m6A modification on the reverse complementary sequences of circ1662, which is essential for the production of circ1662 through a process called intron pairing-driven circularization [38]. Additionally, circRNAs modified with m6A can be recognized by specific reader proteins, potentially leading to changes in their stability and subsequent alterations in their expression levels [30, 39]. Some circRNAs with m6A modification may undergo endoribonucleolytic degradation mediated by the RNase P/MRP complex and requires the cooperative binding of HRSP12 and YTHDF2 proteins [40].

### m6A regulates the function of circRNAs

Due to their unique structure, the translation process of m6A-modified circRNAs differs from that of their parental genes. This mechanism involves the reader protein YTHDF3, which identifies m6A-circRNAs and recruits translation initiation factors, eIF3A and eIF4G2, ultimately triggering cap-independent translation [41].

The m6A modification also affects the role of circRNAs in regulating immunity. CircRNAs without m6A modification can directly activate the Retinoic acid-inducible gene I (RIG-I) signaling pathway to facilitate immune response [42]. In bladder cancer, YTHDF2, an m6A reader, targets DDX58 mRNA, which encodes the RNA helicase RIG-I. The interaction between YTHDF2 and DDX58 mRNA promotes the degradation of RIG-I, a tumor suppressor protein. Elevated levels of YTHDF2 promote bladder cancer progression by inhibiting RIG-I-mediated innate immune signaling. This discovery sheds light on the role of m6A modifications in bladder cancer and highlights the potential impact of targeting YTHDF2 as a therapeutic strategy for improving patient outcomes [43].

### CircRNAs regulate m6A modification

CircRNAs have the ability to bind to m6A writers and erasers, preventing them from modifying other RNAs. This can lead to changes in the m6A modification status of different RNAs, ultimately affecting their expression and function. For example, circ-METTL3 functions as a sponge for the m6A eraser FTO, leading to increased m6A modification and expression of target mRNAs [44]. Besides, circRNAs can interact with proteins that bind to m6A-modified RNAs, influencing the fate of these RNAs in terms of stability, translation, and localization. One notable example is circ-YTHDF1, which interacts with the m6A reader YTHDF1, preventing it from binding to and degrading m6A-modified mRNAs [45]. Furthermore, circRNAs can regulate the expression of genes that encode m6A writers, erasers, and readers, ultimately impacting the overall level of m6A modification in cells. For example, circ-IGF2BP2 controls the expression of the m6A writer METTL3, which eventually affects the overall level of m6A modification in cells [46]. This ability to regulate m6A modification adds another layer of complexity to the regulatory network.

## m6A modification of circRNAs and its role in regulating cancer therapy resistance

**Fig. 4:**
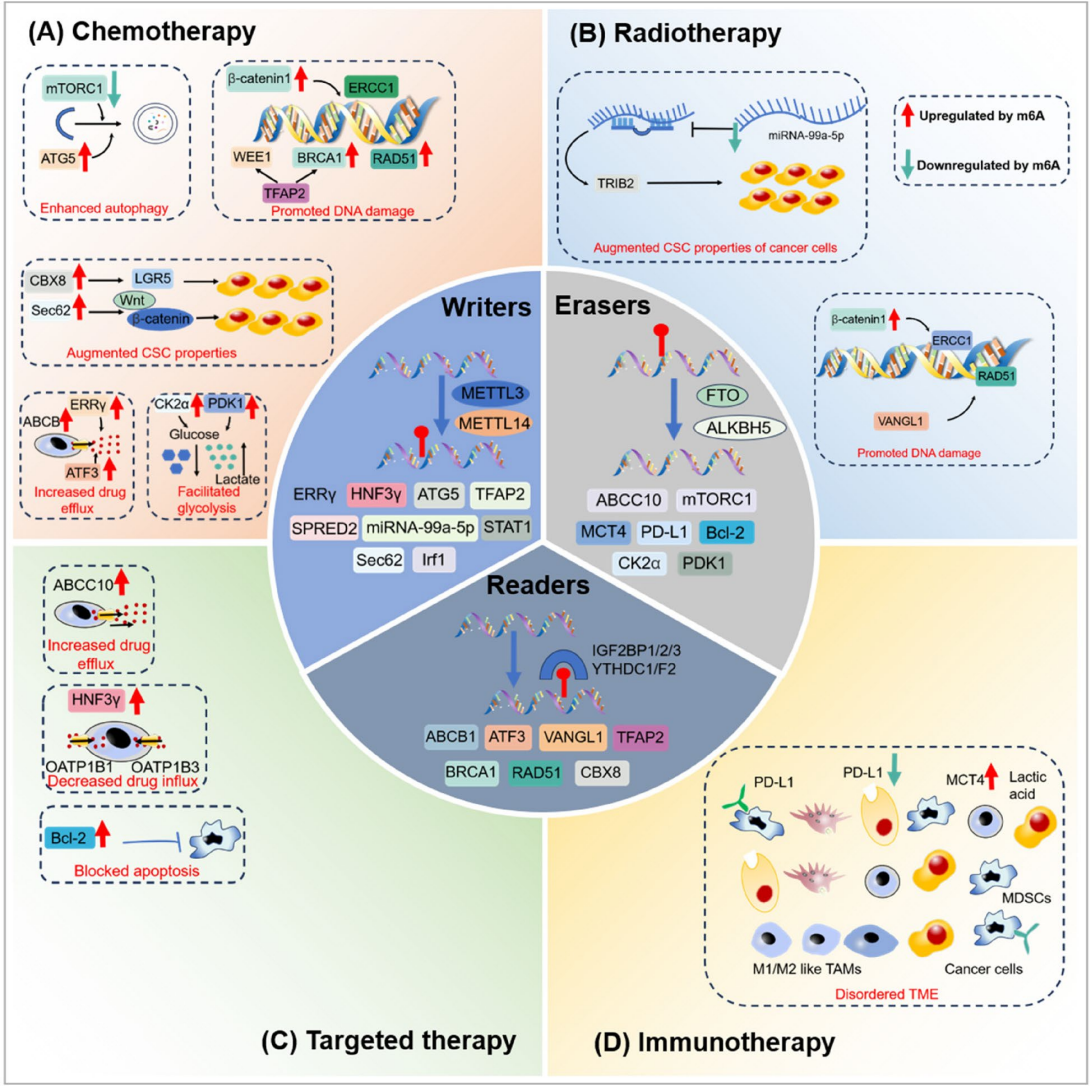
The role of m6A regulators in mediating therapy resistance in cancer. **A** During chemotherapy: m6A modification can hinder the effectiveness of treatment by promoting processes such as glycolysis, DNA damage repair, CSC properties, autophagy, and drug efflux. **B** In radiotherapy: m6A modification can enhance radioresistance by improving DNA damage repair mechanisms and promoting characteristics associated with CSCs. **C** In targeted therapy: m6A modification can increase drug efflux, inhibit cell apoptosis, and impede drug influx, ultimately reducing the efficacy of targeted therapy. **D** In immunotherapy: m6A modification can remodel the tumor microenvironment (TME), thereby contributing to resistance against immunotherapy

In the context of cancer therapeutic resistance, the m6A modification of circRNAs influences the expression and function of genes involved in drug response [47]. Furthermore, m6A modification can affect the interaction between circRNAs and RBPs, leading to changes in their stability and function [48, 49]. The m6A modification of circRNAs has been found to significantly impact the signaling pathways involved in cancer therapy resistance [50], providing new insights into the mechanisms underlying cancer therapeutic resistance [51]. Further investigation is necessary to completely comprehend the precise mechanisms by which m6A modification of circRNAs contributes to this resistance and to examine its potential as a therapeutic target [52]. The increasing knowledge about circRNAs has revealed their crucial functions in the growth, movement, and infiltration of different types of tumor cells. Emerging evidence suggests that circRNAs can impact the sensitivity of cancer treatment through various mechanisms, including regulating drug transportation, DNA repair, cell death, the tumor microenvironment, cellular self-degradation, EMT, cancer stem cells, and glucose metabolism (Table 1). This subsection primarily provides an overview of how m6A modification influences the tumor response to treatment through these various mechanisms (Fig. 4).

**Table 1:** CircRNAs involved in cancer therapy resistance

Therapeutic resistance	Mechanisms	Cancer types	CircRNAs	Roles	Functions	Ref
Chemoresistance	Drug transport	LUAD	circPVT1↑	miR-145-5p↓ → ABCC1↑	Cisplatin/pemetrexed resistance	[53]
		CRC	circ_0007031↑	miR-133b↓ → ABCC5↑	5-FU resistance	[54]
		GC	circMTHFD2↑	miR-124↓ → ABCB1↑	Pemetrexed resistance	[55]
	DNA damage repair	GC	circAKT3↑	PI3K/AKT pathway↑ → BRCA1↑	Cisplatin resistance	[56]
		breast cancer	circSMARCA5↓	SMARCA5↑	Cisplatin resistance	[57]
	Apoptosis	GC	circCCDC66↑	miR-618↓ → BCL-2↑	Cisplatin resistance	[58]
		EC	cDOPEY2↓	CPEB4↑ → MLC-1↑	Cisplatin resistance	[59]
		NSCLC	circ_0002874↑	miR- 1273f↓ → MDM2↑ → P53↓	Paclitaxel resistance	[60]
Radioresistance	TME	GC	circNRIP1↑	miR-138-5p↓ → HIF-1α↑	5-FU resistance	[61]
		NSCLC	circASXL1↑	miR-206↓ → HIF-1α↑	Cisplatin resistance	[62]
	Autophagy	laryngocarcinoma	circPGAM1↑	miR-376a↓ → ATG2A↑	Cisplatin resistance	[63]
		GC	circCUL2↓	miR-138-5p↑ → ROCK2↓	Cisplatin resistance	[64]
		breast cancer	circ_0092276↑	miR-384↓ → ATG7↑	Doxorubicin resistance	[65]
	EMT and CSCs	prostate cancer	circ_0092367↓	miR-1206↑ → ESRP1↓	Gemcitabine resistance	[66]
		NSCLC	circ_0000079↓	FXR1/PRCKI complex↑	Cisplatin resistance	[67]
		NSCLC	circRNA CDR1as↑	HOXA9↓ → miR-641↑	Cisplatin resistance	[68]
		CRC	circ_001680↑	miR-340↓ → BMI1↑	Irinotecan resistance	[69]
	Glycolysis	ESCC	circGOT1↑	miR-606↓ → GOT1↑	Cisplatin resistance	[70]
		prostate cancer	circARHGAP29↑	c-Myc↑ → LDHA↑	Docetaxel resistance	[71]
		neuroblastoma	circDLGAP4↑	miR-143↓ → HK2↑	Doxorubicin resistance	[72]
		NSCLC	circ_0008928↑	miR-488↓ → HK2↑	Cisplatin resistance	[73]
Radioresistance	TME	HCC	cZNF292↑	SOX9 nuclear translocation↑ → Wnt/β-catenin pathway↑	Radioresistance	[74]
	Glycolysis	breast cancer	circABCB10↑	miR-223-3p↓ → PFN↑	Radioresistance	[75]
Targeted therapy resistance	Drug transport	NSCLC	circSETD3↑	miR-520 h↓ → ABCG2↑	Gefitinib resistance	[76]
	Autophagy	CML	circ_0009910↑	miR-34a-5p↓ → ULK1↑	Imatinib resistance	[77]
	EMT and CSCs	PC	circ_0013587↓	miR-1227↑ → E-cadherin↓	Erlotinib resistance	[78]
Immunotherapy resistance	TME	ICC	circHMGCS1–016↑	miR-1236–3↓ → CD73/ GAL-8↑	Anti-PD-1 therapy resistance	[79]
		HNSCC	circFAT1↑	STAT3↑ → CD8 + T cells infiltration↓	Anti-PD-1 therapy resistance	[80]

### Effects of m6A modification on glycolysis, DNA damage repair, CSC properties, autophagy, and drug efflux during chemotherapy

#### m6A-induced alterations in glycolysis

Researchers have now identified disrupted energy metabolism as a defining characteristic of cancer. In 1924, Otto Warburg discovered that tumor cells primarily rely on glycolysis for energy production, even in the presence of oxygen [81]. This phenomenon, known as "aerobic glycolysis" or the "Warburg effect", involves rapid glucose consumption by tumor cells to generate lactic acid and ATP. While glycolysis yields less ATP than oxidative phosphorylation, it is crucial for the rapid proliferation of cancer cells due to its faster energy production rate [82]. Additionally, the abundant lactic acid produced by tumor cells contributes to an acidic TME. Furthermore, glycolytic intermediates can act as building blocks for synthesizing essential biomolecules, which play a critical role in driving tumor growth, metastasis, and treatment resistance [83]. Recent research conducted by Yu's group has revealed that the depletion of ALKBH5 results in an increase in the expression of casein kinase 2α (CK2α). This finding suggests that the m6A modification promotes cisplatin resistance in bladder cancer (BC) by upregulating the CK2α-mediated glycolysis pathway [84].

#### m6A-induced alterations in DNA damage repair

The field of m6A modification of circRNAs in DNA damage repair is rapidly expanding. The m6A modification regulates the expression and functions of circRNAs in DNA damage repair processes [85]. This modification regulates glycolysis efficiency and promotes oncogenesis [86]. Furthermore, m6A modification in circRNAs is linked to various physiological processes, such as DNA damage repair [87]. However, more research is needed to fully elucidate the role of m6A modification of circRNAs in DNA damage repair [88].

Several proteins involved in repairing DNA damage, such as ERCC1, have been identified as crucial factors in the resistance to chemoradiotherapy. These proteins are closely linked to m6A modification. ERCC1, located on chromosome 19, plays a vital role in the nucleotide excision repair (NER) pathway [89]. Higher levels of ERCC1 correlate with cisplatin resistance in various cancers, including gastric (GC) and cervical cancers [90]. To better understand the role of FTO in chemoradiotherapy resistance, Zhou et al. investigated its expression and discovered its regulatory function in both cisplatin and radiotherapy resistance. Their research showed that FTO increases the expression of β-catenin, which in turn upregulates ERCC1. This upregulation of ERCC1, through NER activation, leads to treatment failure in cervical squamous cell carcinoma (CSCC) [91]. Double-strand breaks (DSBs) are the most lethal form of DNA damage, but fortunately, cells have the ability to repair them through homologous recombination (HRR) [92]. However, abnormal expression of essential genes involved in HRR can alter the sensitivity of tumor cells to cancer therapy. This concept has been illustrated in several studies. For example, reduced expression of epithelial membrane protein 3 (EMP3) leads to increased levels of YTHDC1, which promotes DNA repair in breast cancer cells by upregulating BRCA1 and RAD51, ultimately resulting in chemoresistance [93]. Interestingly, IGF2BP1-mediated stabilization of TFAP2C in cisplatin-resistant seminoma cells contributes to the activation of WEE1 and BRCA1, further promoting DNA repair [94].

A previous study has shown that the m6A modification of circRNA circNSUN2 increases its stability and enhances the recruitment of DNA damage repair proteins to sites of DNA damage. This circRNA acts as a scaffold to promote the assembly of DNA repair complexes, ultimately enhancing efficient DNA damage repair [95]. Another study has revealed that the m6A modification of circRNA circAmotl1a regulates the expression of DNA damage response genes by interacting with specific RBPs [96]. Additionally, the m6A modification of circHIPK3 promotes its interaction with the DNA damage repair protein PARP1, which is involved in single-strand break and base excision repair [97]. Furthermore, the m6A modification of circDENND4C suppresses its interaction with the DNA damage repair protein ATM [98], thereby affecting the recruitment of DNA repair factors to DNA damage sites and altering the efficiency of DNA damage repair [99].

These findings indicate that m6A modification of circRNAs plays a crucial role in regulating DNA damage repair processes [93]. By modulating the expressions and functions of circRNAs, m6A modification can influence the efficiency and accuracy of DNA damage repair, thereby maintaining genomic stability [100]. Additionally, more research is necessary to identify the precise mechanisms by which m6A modification influences circRNA-mediated DNA damage repair. Furthermore, it is important to explore the potential implications of DNA damage in diseases such as cancer and neurodegenerative disorders [101, 102].

#### The impact of circRNA m6A on EMT and CSCs

Recent studies have revealed that the modification of circRNAs through m6A can play a crucial role in regulating EMT and CSCs [103], ultimately influencing cancer progression [104]. EMT is a process closely associated with increased invasiveness and metastasis in cancer [105]. The impact of m6A modification on EMT-related circRNAs and their effect on cancer progression is currently a topic of active research.

Firstly, m6A modification can increase the stability of circRNAs, resulting in higher levels of expression [106]. These increased expressions of certain circRNAs are associated with EMT and CSC properties [107]. Furthermore, circRNAs are identified as promoting EMT by serving as miRNA sponges or binding to specific proteins, thereby regulating the expression of genes related to EMT [108].

Secondly, m6A modification can potentially regulate the function of circRNAs in EMT and CSCs. Studies have shown that m6A modification can influence the binding ability of circRNAs to specific proteins or miRNAs, thereby affecting their regulatory roles in EMT and CSCs [109]. For example, m6A modification of circRNA can alter its interaction with RBPs, resulting in changes to downstream signaling pathways involved in EMT and CSC maintenance [110]. Furthermore, circRNAs play a significant role in EMT-related processes such as cell invasion, metastasis, and the regulation of EMT-related transcription factors and signaling pathways [111]. Additionally, m6A modification of the circRNA circZEB1 promotes EMT in GC cells by increasing the expression of the EMT transcription factor ZEB1 [112]. Moreover, m6A modification encourages the acquisition of CSC properties by activating the Wnt/β-catenin signaling pathway, leading to chemoresistance in colorectal cancer (CRC) [113].

In the context of CSCs, circRNAs have been implicated in their functions and potential applications. Exosome-associated circRNAs have also been identified as critical regulators of EMT in cancer [114]. Additionally, m6A modification affects the expression of genes involved in EMT and CSCs [115]. m6A modification on mRNA transcripts can affect their stability, translation, and splicing, ultimately regulating the genes related to EMT and CSCs [116]. m6A modification of circSOX2 promotes CSC self-renewal and tumorigenicity in colorectal cancer by increasing SOX2 expression [117]. Consistently, m6A modification of the circCD44 promotes CSC self-renewal and tumorigenicity in glioblastoma by upregulating the expression of the stem cell marker CD44 [118]. This modulation of gene expression has also been linked to regulating the EMT process and maintaining CSC properties [119].

In summary, the m6A modification of circRNAs is identified as a crucial regulatory mechanism in the processes of EMT and CSCs [120]. Understanding the impact of circRNA m6A modification on EMT and CSCs can offer valuable insights for developing new therapeutic approaches in tumor treatment [121]. As research in this area progresses, we can expect to gain a deeper understanding of the specific mechanisms by which m6A modification regulates EMT and CSCs, and how this regulation can be utilized for therapeutic purposes [122].

#### m6A modification of circRNAs regulates autophagy

Recent research has shown that m6A modification is crucial in activating autophagy and forming autophagosomes. A study conducted by Li et al. demonstrated that overexpression of YTHDF1 under hypoxia facilitated the translation of ATG2A and ATG14, resulting in the activation of autophagy [123]. Moreover, the activation of autophagy during hypoxia can lead to drug resistance in various types of tumor cells. This is attributed to the function of FTO in decreasing levels of pro-survival autophagy, rendering gastric cancer cells more susceptible to chemotherapy through the mTORC1 pathway [124]. METTL3 also plays a role in promoting autophagy, resulting in decreased sensitivity of seminoma cells to cisplatin [125].

Furthermore, m6A modification of circRNAs impacts the interaction between circRNAs and miRNAs. miRNAs are small RNA molecules responsible for regulating gene expression at the post-transcriptional level. CircRNAs can bind to miRNAs and inhibit their activity, acting as sponges for miRNAs. The m6A modification of circRNAs can be altered in their binding affinity to miRNAs, thereby modulating the regulation of miRNA targets and ultimately impacting autophagy-related pathways [126]. In summary, the m6A modification of circRNAs represents a novel mechanism by which circRNAs can regulate autophagy [127]. Additional experimental studies are needed to identify the specific circRNAs, uncover the mechanisms of m6A modification in regulating autophagy, and determine the implications for various cellular processes and diseases [11, 128].

#### m6A-induced alterations in drug transport

The m6A modification significantly affects the resistance of cancer therapy through various mechanisms, such as the regulation of gene expression and the alteration of protein function [129]. One of these mechanisms is the alteration of drug transport [130]. Additionally, the m6A modification plays an essential role in the expression of the ABC transporter family, which strongly contributes to multidrug resistance [131].

Resistance to cancer drugs is often caused by enhanced drug efflux and reduced drug influx. A recent study has identified a new m6A binder, insulin-like growth factor 2 mRNA-binding protein 3 (IGF2BP3), which regulates gene expression by improving the stability and nuclear export of specific mRNA targets [132]. Overexpression of IGF2BP3 interacts with the m6A site of P-gp, leading to increased expression of P-gp and reduced sensitivity of colorectal cancer cells to therapy [133]. Moreover, m6A modification-induced upregulation of ERRγ promotes chemoresistance by increasing P-gp levels. Interestingly, Liu et al. found that YTHDF2 regulates the expression of ATF3, a transcription factor that interacts with the enhancer region of ABCB1 and promotes its expression, resulting in tamoxifen resistance [134].

### The impact of m6A modification on DNA damage repair and CSC characteristics in radiotherapy

#### m6A modification improves DNA damage repair in radiotherapy

Cells have the ability to repair DNA damage caused by radiotherapy. A study on lung adenocarcinoma (LUAD) found that increased levels of m6A and decreased levels of miR-29b-3p resulted in an upregulation of VANGL1. This activation of VANGL1 triggers the BRAF/TP53BP1/RAD51 pathway, promoting DNA repair and protecting cells from radiation damage [135].

#### m6A modification augments CSC characteristics in radiotherapy

Recent studies have shown that the modification of circRNAs through m6A has an impact on radiotherapy, thereby influencing cancer progression. For example, a study conducted by Liu et al. identified the METTL14/miR-99a-5p/TRIB2 axis as a significant contributor to the characteristics of CSCs and their resistance to radiotherapy in esophageal squamous cell carcinoma (ESCC). The positive correlation between this axis and these aggressive features suggests potential therapeutic targets for ESCC treatment [136].

### The potential of m6A modification to affect drug efflux, cell apoptosis, and drug influx in targeted therapy

#### m6A modification increases drug efflux in targeted therapy

Apart from chemotherapy, m6A modification also occurs in targeted therapy. For example, Gefitinib, a drug commonly used to treat non-small cell lung cancer (NSCLC), can be effluxed by the transporter protein ABCC10, thereby impacting its intracellular concentration inside the cell [137]. Interestingly, research has shown that FTO, an enzyme enriched in serum exosomes from patients resistant to gefitinib, can increase the expression of ABCC10 through m6A modification. This suggests that overexpression of FTO may contribute to the development of gefitinib resistance in NSCLC [138].

#### m6A modification induces cell apoptosis alterations in targeted therapy

Apoptosis, a programmed cell death process, is crucial in normal development and disease progression [139]. Evading apoptosis is a hallmark of cancer and a major cause of treatment failure. Key regulators of apoptosis include the BCL-2 family proteins. The BCL-2 family consists of both pro-apoptotic members, such as BAX and BAK, which trigger cell death, and anti-apoptotic members, such as BCL-2 and MCL-1 [140]. m6A modifications can influence the expression of these BCL-2 family proteins. For example, METTL3, an m6A writer enzyme, increases BCL-2 expression in breast cancer, thereby suppressing apoptosis [141]. This finding is particularly relevant because high levels of BCL-2 are linked to resistance to multiple drugs, including paclitaxel, tamoxifen, and trastuzumab [142, 143].

#### m6A modification impedes drug influx in targeted therapy

In addition to the ABC efflux transporter family, the m6A modification also affects the expression of specific transporters, such as organic anion-transporting polypeptides (OATP) [144]. The OATP transporter family consists of 11 members. Notably, OATP1B1 and OATP1B3 are located on the basement membrane of human liver cells (hepatocytes) and responsible for transporting drugs within the liver. Sorafenib, a targeted therapy for inoperable or advanced hepatocellular carcinoma (HCC) and clear cell renal cell carcinoma (ccRCC), is known to be a substrate for both OATP1B1 and OATP1B3. A recent study revealed that HCC tissues have lower levels of hepatocyte nuclear factor 3γ (HNF3γ) compared to healthy neighboring tissues. This decrease was linked to reduced METTL14 expression, contributing to sorafenib resistance by suppressing OATP1B1 and OATP1B3 expression [145].

### The impact of circRNA m6A regulators on the tumor microenvironment in immunotherapy

The TME comprises various cell types, including immune cells, tumor cells, and stromal cells [146]. These cells interact with each other, creating a microenvironment that promotes tumor growth and survival [31]. The m6A modification of circRNAs is crucial in remodeling the TME by regulating gene expression involved in tumor progression and metastasis [147, 148]. By targeting specific circRNAs, m6A regulators can influence the expression of genes involved in the TME, such as immune checkpoint molecules, cytokines, and chemokines [32, 149]. This can result in either an enhanced immune response against tumors or immune evasion by tumor cells [150]. Consistently, circRNA m6A regulators can also influence the interaction between cancer cells and stromal cells in the TME [151]. Furthermore, m6A modification can regulate the expression of extracellular matrix components and signaling molecules involved in cell–cell communication, ultimately impacting tumor cell invasion, angiogenesis, and metastasis [152]. Apart from regulating gene expression, m6A modification of circRNAs can also influence the interaction between circRNAs and proteins that regulate the TME [153]. The potential for using circRNA m6A regulators to remodel the TME as a target for cancer treatment has been identified [154]. However, research on the role of m6A modification of circRNAs in remodeling the TME is still in its early stages [155], but it has shown potential for regulating immune responses in cancer.

## The relationship between m6A modification and circRNAs in cancer therapy resistance

**Fig. 5:**
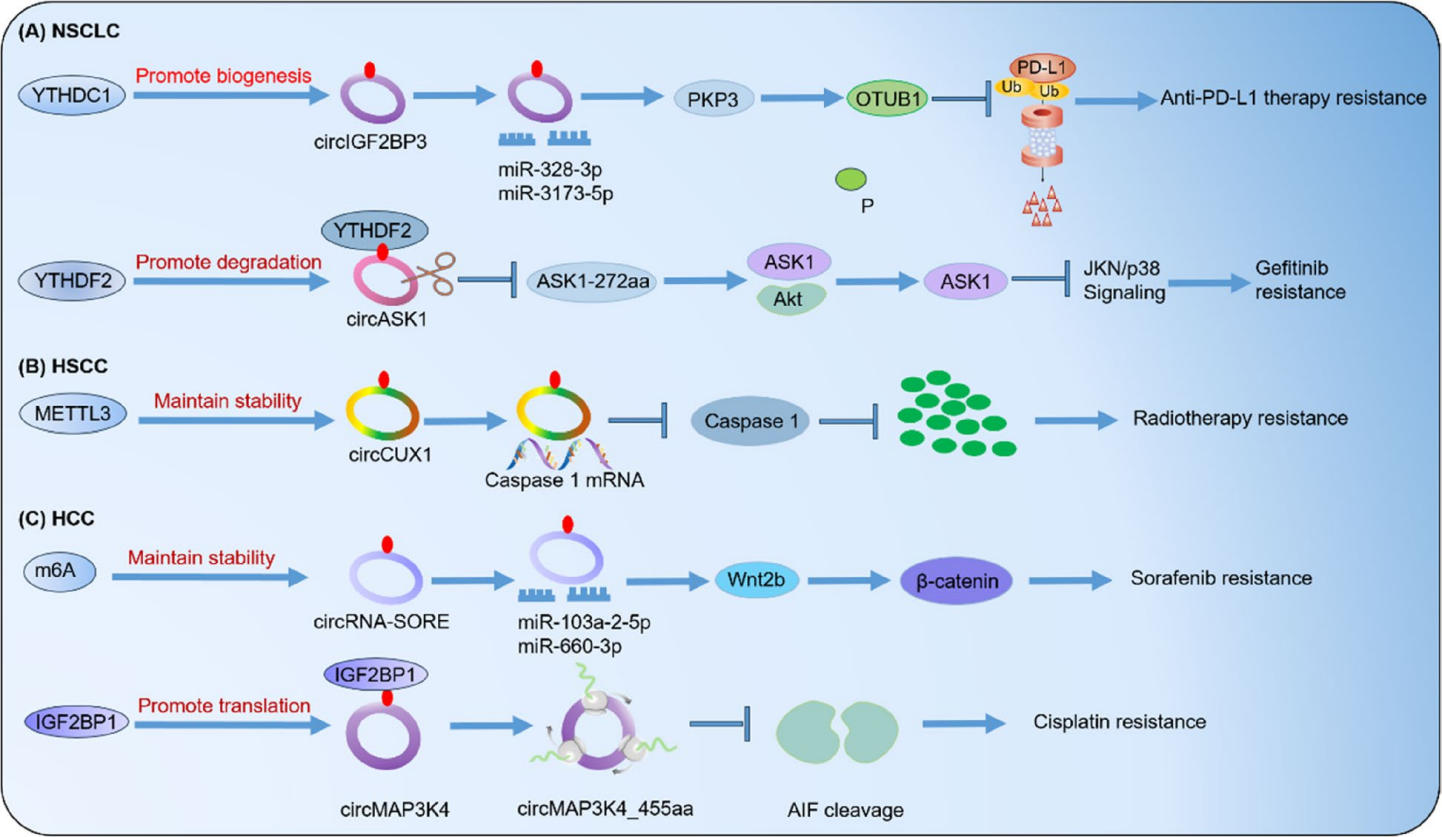
The role of m6A-modified circRNAs in cancer therapy resistance. m6A modification significantly impacts the biogenesis, translation, and degradation of circRNAs, ultimately affecting their role in therapeutic resistance in various types of cancer. **A** In non-small cell lung cancer (NSCLC), YTHDC1 and YTHDF2 play a crucial role in facilitating anti-PD-L1 therapy and gefitinib resistance by increasing the biogenesis of circIGF2BP3 and degrading circASK1, respectively. **B** In head and neck squamous cell carcinoma (HSCC), METTL3 increases the stability of circCUX1, leading to radioresistance. **C** In hepatocellular carcinoma (HCC), m6A regulators promotes the stability of circRNA-SORE and the translation of circMAP3K4, resulting in resistance to sorafenib and cisplatin, respectively

In this review, we provide a concise overview of the recent studies that have examined the regulatory role of m6A-modified circRNAs in the development of treatment resistance in various types of cancers, including hepatocellular carcinoma (HCC), hypopharyngeal squamous cell carcinoma (HSCC), and non-small cell lung carcinoma (NSCLC) (Table 2, Fig. 5).

**Table 2:** m6A-modified circRNAs in cancer therapy resistance

Cancer types	M6A regulators	Roles of m6A in circRNAs	Functions	Mechanisms	Ref
HSCC	METTL3	Promotes m6A modification of circCUX1 to stabilizes its expression	Radiotherapy resistance	Decreases the release of inflammatory factors in TME	[30]
HCC	m6A	Elevates circRNA-SORE expression via increasing RNA stability	Sorafenib resistance	Activates the Wnt/β-catenin pathway	[156]
	IGF2BP1	Promotes m6A-modified circMAP3K4 translation	Cisplatin resistance	Inhibits apoptosis	[157]
NSCLC	YTHDC1	Facilitates the biogenesis of m6A-modified circIGF2BP3	Anti-PD-L1 therapy resistance	Promotes tumor immune evasion	[158]
	YTHDF2	Increases m6A-modified circASK1 degradation	Gefitinib resistance	Represses apoptosis	[159]

### Hypopharyngeal squamous cell carcinoma (HSCC)

Improving the diagnosis of HSCC requires identifying the molecular mechanisms that promote therapy resistance. A previous study found that the expression of circ_0058106 is upregulated in HSCC tissues. Circ_0058106 is reported to regulate the Wnt2b/β-catenin/c-Myc pathway, thereby promoting tumorigenesis and EMT in HSCC [160]. Similarly, another study showed that upregulated circMATR3 enhances proliferation and invasion, while inhibiting apoptosis [161]. It is plausible that m6A modification plays a regulatory role in the expression levels of circRNAs. This hypothesis is supported by a recent study by Wu et al., which found that METTL3-mediated m6A methylation stabilizes the expression of circCUX1. CircCUX1, in turn, interacts with caspase 1, leading to a decrease in its expression and a subsequent reduction in IL-1β levels in the tumor microenvironment. This may potentially contribute to the development of tolerance in HSCC [30].

### Hepatocellular carcinoma (HCC)

Recently, several studies have highlighted the crucial role of the interaction between circRNAs and m6A modification in various aspects of HCC [162, 163]. For example, it is found that METTL3 promotes the production of circHPS5 in HCC. The m6A-regulated circHPS5 functions as a sponge for miR-370, increasing HMGA2 expression and promoting EMT, ultimately facilitating the progression of HCC [164]. Sorafenib, a commonly prescribed targeted drug for HCC, is hindered in its therapeutic effectiveness by the development of acquired resistance. In sorafenib-resistant HCC, the elevated levels of circRNA-SORE, transported through exosomes, impede the degradation of YBX1 by interacting with it, thereby reducing the drug's therapeutic effectiveness [165].

### Non‑small‑cell lung cancer (NSCLC)

Lung cancer is a significant threat to human well-being and is one of the leading causes of cancer-related deaths worldwide [157]. In recent years, targeted therapy and immunotherapy have made promising advancements, providing hope for patients with lung cancer. However, a major challenge remains in the development of acquired resistance to these treatments. Mettl3 plays a crucial role in promoting the circularization of circIGF2BP3 through its m6A modification. This circular RNA acts as a competitive endogenous RNA (ceRNA), sequestering miR-328-3p and miR-3173-5p to increase the expression of PKP3. PKP3 interacts with the RNA-binding protein FXR1, stabilizing OTUB1 mRNA and promoting the abundance of PD-L1 through deubiquitination. Importantly, deletion of tumor PD-L1 abolishes the effect of the circIGF2BP3/PKP3 axis on CD8^+^ T cell response. In a Lewis lung carcinoma mouse model, inhibition of the circIGF2BP3/PKP3 pathway enhanced the efficacy of anti-PD-1 therapy. Furthermore, the PKP3/PD-L1 signature and the level of infiltrating CD8^+^ T cells can be used to classify NSCLC patients into distinct risk groups [166]. It has been confirmed that IGF1R promotes proliferation by activating the PI3K/AKT pathway, leading to osimertinib resistance [158]. Overall, understanding the mechanisms behind acquired resistance in NSCLC and exploring the role of circRNAs, such as circIGF2BP3 and hsa_circ_0005576 [167, 168], can provide valuable insights for developing more targeted and effective treatment strategies [169].

Furthermore, studies have demonstrated that increased levels of circNDUFB2 can effectively impede the proliferation and dissemination of NSCLC cells. This is achieved through circNDUFB2 as a scaffold, facilitating the interaction between TRIM25 and IGF2BPs, a protein known to drive tumor progression and metastasis. The formation of the TRIM25/circNDUFB2/IGF2BPs complex results in the tagging and degradation of IGF2BPs, a process further enhanced by the m6A modification of circNDUFB2. Additionally, circNDUFB2 can activate the RIG-I-MAVS signaling pathway by binding to RIG-I, attracting immune cells to the TME [170].

### CircRNA influences m6A modification in cancer therapy resistance

**Fig. 6:**
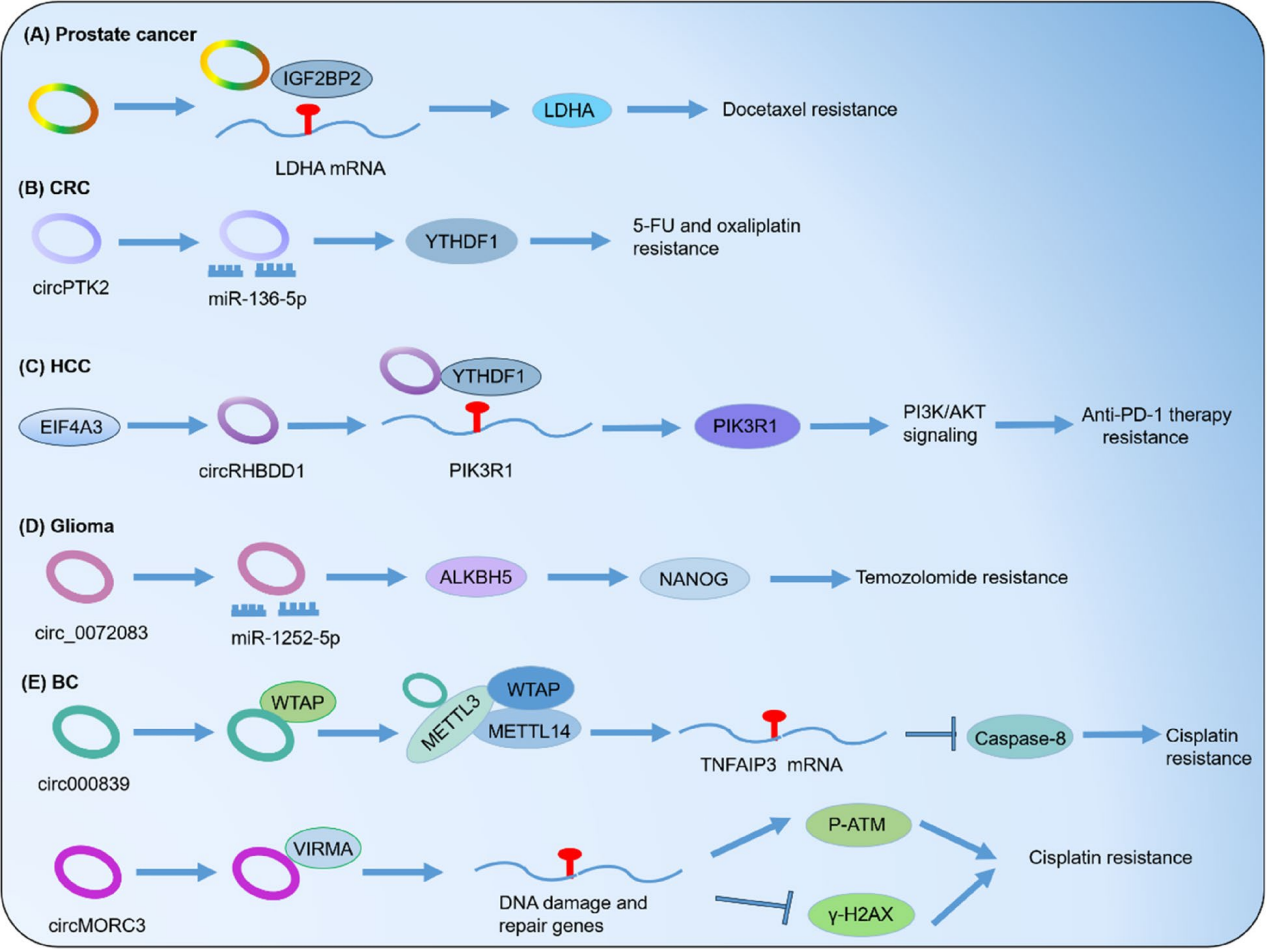
CircRNAs influence m6A modification in cancer therapy resistance. CircRNAs can either modulate the expression of m6A regulators or interact with them, significantly impacting m6A functions in various types of cancer. **A** In prostate cancer, circARHGAP29 interacts with IGF2BP2, leading to an increase in docetaxel resistance. **B** In colorectal cancer (CRC), circPTK2 increases the level of YTHDF1, resulting in resistance to 5-FU and oxaliplatin. **C** In hepatocellular carcinoma (HCC), circRHBDD1 recruits YTHDF1, leading to resistance to anti-PD-1 therapy. **D** In glioma, circ_0072083 promotes the expression of ALKBH5, resulting in resistance to temozolomide. **E** In breast cancer (BC), circ0008399 facilitates the formation of MTC by interacting with WTAP, leading to an increase in cisplatin resistance. Additionally, circMORC3 promotes cisplatin resistance by interacting with VIRMA

Notably, abnormal circRNA expression affects m6A modification in cancer. We have compiled a summary of studies on m6A regulation by circRNAs, including CRC, BC, HCC and glioma, as shown in Table 3 and Fig. 6.

**Table 3:** CircRNAs related to m6A modification in different types of cancer

Cancer	circRNAs	Roles of circRNAs in m6A	Functions	Mechanisms	Ref
Glioma	circ_0072083	Promotes ALKBH5 expression via sponging miR-1252-5p	Temozolomide resistance	Maintains glioma stem cells	[171]
CRC	circPTK2	Elevates YTHDF1 level by targeting miR-136-5p	5-FU/oxaliplatin resistance	–	[172]
HCC	circRHBDD1	Recruits YTHDF1	Anti-PD-1 therapy resistance	Elevates glycolysis	[173]
Bladder cancer	circ0008399	Facilitates the formation of MTC through combining with WTAP	Cisplatin resistance	Boosts anti-apoptosis	[174]
	circMORC3	Interacts with VIRMA and elevates global m6A level	Cisplatin resistance	Promotes DNA repair and suppresses DNA damage	[175]
Prostate cancer	circARHGAP29	Interacts with IGF2BP2	Docetaxel resistanc	Promotes glycolysis	[71]

### Potential challenges associated with targeting m6A writers, readers, and erasers for therapeutic benefit

Small molecules are crucial tools for understanding the roles of specific RNA modifications in certain types of cancer. Table 4 summarizes small molecule inhibitors that target writers, readers, and erasers of m6A modifications.

**Table 4:** Small-molecule inhibitors targeting RNA modifications

Target gene	Inhibitor name	Cancer	Anticancer potency
METTL3/METTL14	STM2457	AML	0.6–10.3 μM
	STC-15	Advanced tumors	
	Multiple small molecules	AML, ovarian adenocarcinoma	80 nM–1.39 μM
	Multiple small molecules	AML	< 1 μM, < 10 μM
	UZH2	AML	12 μM, 70 μM
	UZH1a	prostate cancer	Not tested
	CDIBA	AML	13–22 μM
	Eltrombopag	osteosarcoma	8.28 μM
	Elvitegravir	AML	Reduced metastasis
YTHDF	Ebselen	Prostate cancer	26.83 μM
YTHDF1	Salvianolic acid (SAC)	Neuronal tissue	N/A
YTHDF2	CpG-siRNA^YTHDF2^	Mouse melanoma	Reduced tumor growth
FTO	CS1	AML	22–753 nM
	FB23-2	AML	1.9–5.2 μM
	MO-I-500	Triple-negative Breast cancer	Inhibited survival and colony formation
	MA	Hela	N/A
	MA2	GSCs	Inhibited growth and self-renewal of GSCs
	FTO-04	GSCs, glioblastoma	Impaired self-renewal properties
	Dac51	Melanoma	Inhibited tumor growth
	R-2HG	Glioma	Decreased cell proliferation
ALKBH5	2-[(1-hydroxy-2-oxo-2-phenylethyl)sulfanyl]acetic acid, 4-{[(furan-2-yl)methyl] amino}-1,2-diazinane-3,6-dione	AML	1.38–47.8 μM
PUS7	C17	Glioblastoma	56.77–177.4 nM

*Abbreviations*: *ALKBH5 *AlkB homolog 5, *AML *Acute myeloid leukemia, *FTO *Fat mass and obesity-associated protein, *GSCs *Glioma stem-like cells, *IDH *Isocitrate dehydrogenases, *MA *Meclofenamic acid, *METTL3 * Methyltransferase-like 3, *METTL14 *Methyltransferase-like 14, *N/A *Not available, *PUS *Pseudouridine synthase, *YTHDF *YTH domain-containing family protein

Despite promising preclinical data, targeting dysregulated m6A modifiers with small-molecule inhibitors remains challenging. A deeper understanding of the role and underlying mechanisms of m6A machinery in cancer is crucial for selecting effective therapeutic targets. In addition to small molecules, protein degraders offer an alternative strategy for targeting m6A readers. Combinatorial targeting of multiple oncogenic m6A modifiers shows promise for achieving optimal therapeutic outcomes. For example, simultaneously targeting writers and erasers could have a synergistic effect, considering their distinct tumorigenic mechanisms. Functional studies using genetic depletion models and combinatorial genetic deletions can validate synergy and guide researchers in developing novel combination therapies. Due to the context-dependent nature of m6A modifiers, different cancer types or subtypes may require unique combination therapies. Future possibilities may include CRISPR-based manipulation of specific m6A modifications critical for cancer development. The complex and interactive nature of cancer and the TME suggests that targeting oncogenic m6A modifiers will likely require a combination with other therapies to achieve curative effects.

Developing effective RNA-based therapies and targeting RNA-modifying proteins requires optimizing drug discovery pipelines. Current drugs targeting these enzymes often cause off-target effects, hindering their clinical potential. Overcoming the challenge of off-target effects and ushering in a new era of targeted RNA-based interventions can be achieved through advancements in computational models, high-throughput enzymatic assays, and exploration of interconnected pathways.

Naturally, RNA modifications can enhance the stability, efficacy, and specificity of therapeutic RNAs. We can optimize these benefits, such as increased stability, improved efficacy, and enhanced specificity, through strategic natural and synthetic modifications targeting specific sequence contexts and locations. Integrating comparative single-cell analyses with advanced molecular, biochemical, and cellular studies in healthy and diseased human cells can fully harness the potential of RNA modifications for clinical applications.

## Exosomal circRNA functions as clinical biomarker

A compilation of the most recent discoveries exploring circRNAs and exosomal circRNAs as potential diagnostic or prognostic indicators for different cancer types can be found in Table 5. As more experimental data is gathered, we are optimistic that exosomal circRNAs will prove to be highly promising cancer biomarkers and eventually have practical applications in clinical settings.

**Table 5:** The clinical applications of circRNAs and exosomal circRNAs as biomarkers for cancer diagnosis or prognosis

Registration number/NCT number	Study type	Study phase	Recruiting status	Tumor type	Sample name	Sample size
ChiCTR2300069863	Observational study	0	Recruiting	Cholangiocarcinoma	Bile, serum	320
ChiCTR1900027419	Basic science	0	Not yet recruiting	Lung cancer	Blood	58
ChiCTR1900024188	Diagnostic test	0	Recruiting	Prostate cancer	Urine	300
ChiCTR1800019529	Diagnostic test	Diagnostic new technique clinical study	Recruiting	Prostate cancer	Plasma	200
ChiCTR1800018038	Diagnostic test	Diagnostic new technique clinical study	Not yet recruiting	Pancreatic cancer	Blood	20
NCT05771337	Observational study	Not available	Not yet recruiting	Breast cancer	Blood	80
NCT04584996	Observational study	Not available	Recruiting	Pancreatic cancer, biliary tract cancer	Blood, bile	186
NCT04464122	Observational study	Not available	Recruiting	Neuroendocrine neoplasm	Not available	60
NCT03334708	Observational study	Not available	Recruiting	Pancreatic cancer	Blood	700

CircRNAs boast exceptional stability due to their unique covalent closed-loop structure, which protects them from exonuclease degradation. This results in a significantly longer lifespan compared to their parent mRNAs, while mRNAs typically degrade within 10 h, circRNAs can persist for up to 48 h [176]. These advantages make circRNAs a promising tool for cancer diagnosis and prognosis prediction. However, the low abundance of exosomal circRNAs poses a challenge in accurately detecting and identifying them in body fluids using current methods [177]. Furthermore, the cost of detecting circRNAs in tissue or exosomes is higher than that of existing tests. Therefore, advanced techniques are needed to improve the accuracy and affordability of detecting exosomal circRNAs before they can be effectively used in lipid biopsies for clinical applications. Combining exosomal circRNAs with other exosomal cargoes or current biomarkers may provide a more precise and comprehensive assessment for early cancer diagnosis and predicting clinical outcomes.

Although ultrasensitive flow cytometry has been widely used to identify the origin of external vesicles by staining cell-specific markers, the classification of exosomes needs to be clarified and consistent across different studies [178]. Furthermore, the accuracy of using exosomal circRNAs as diagnostic and prognostic biomarkers for human malignancies may be affected by the choice of circRNA profiling method. Techniques such as RNA-seq, microarray, and qRT-PCR can each impact the results [179]. Therefore, standardized protocols for exosome isolation, purification, characterization, and profiling methods are urgently needed in exosomal circRNA research.

## Conclusions and future prospects

The development of resistance to cancer treatment presents significant challenges, such as higher mortality rates and unfavorable prognoses for cancer patients. Therefore, it is imperative to thoroughly investigate the potential molecular mechanisms that contribute to therapeutic resistance in cancer. While many studies have proposed various factors that can impact the responsiveness of tumor cells to treatment, the exact mechanisms responsible for therapeutic resistance still need to be fully understood. In this comprehensive review, we have synthesized the latest findings on the interplay between m6A modification and circRNAs, and their implications for therapeutic resistance. This review offers valuable insights for future research on overcoming therapeutic resistance in cancer.

Epigenetic regulation, specifically modifications in RNA, can significantly impact post-transcriptional gene expression. This dysregulation includes alterations in ABC transporters, OATP transporters, genes involved in autophagy, DNA damage and repair genes, all of which contribute to cancer relapse. However, the extent to which these epigenetic mechanisms contribute to the dysregulation of circRNAs. Our research has revealed that m6A modifications significantly influence the biosynthesis, localization, translation, and degradation, potentially leading to abnormal circRNA expression. However, only a few m6A-modified circRNAs have been identified as being associated with the response to tumor therapy.

Improving the detection of m6A modification presents a significant technical challenge. While several techniques have been developed in recent years, such as methylated RNA immunoprecipitation sequencing (MeRIP-seq), m6A labeling-based sequencing (m6A-label-seq), and methylation-iCLIP (miCLIP), they all have limitations and require further refinement. For instance, MeRIP-seq relies on antibodies, which can lead to false-positive results due to their lack of specificity. Similarly, although m6A-label-seq can recognize m6A sites with single-base resolution, it is limited in its ability to detect a large number of m6A residues [180–183]. Furthermore, there is growing evidence to suggest that other RNA modifications, such as pseudouridine (Ψ) and 5-methylcytosine (m5C), are prevalent in non-coding RNAs, including ribosomal RNAs, miRNAs, and lncRNAs [184]. Therefore, it is hypothesized that circRNA levels may be regulated not only by m6A modifications but also by other types of RNA modifications.

Currently, scientists have discovered various natural compounds that target m6A readers, writers, and erasers. For example, Saikosaponin D (SsD) increases the overall m6A modification level by inhibiting the m6A demethylase FTO. This, in turn, can alleviate leukemia resistance to TKIs therapy by reducing the stability of BCL-2 [174]. In addition to SsD, other substances such as fusaric acid, curcumin, STM2457, and chidamide can modulate m6A regulatory proteins [185]. The discovery of these modulators that target m6A offers new possibilities for overcoming therapeutic resistance.

Recently, numerous studies have revealed that circRNAs are highly abundant in exosomes. These exosomes facilitate the progression of cancer by acting as vehicles for transporting molecules, particularly circRNAs. These exosomes are present in various bodily fluids, including urine, blood, and saliva. Consequently, the potential of exosomal circRNAs as valuable markers for cancer prognosis in liquid biopsies should not be disregarded [186]. To fully realize this potential, the development of highly accurate detection techniques is crucial [42]. Certain circRNAs can be to be translated through IRES and m6A-dependent pathways [187]. Additionally, certain circRNAs possess the capacity to regulate the expression of m6A regulators through their ability to sponge miRNAs and interact with m6A writers/readers/erasers. However, it is still unclear whether circRNAs can impact m6A modification through other biological functions, such as gene transcription regulation or translation, remains unclear.

In summary, m6A modification, circRNAs, and their interactions are crucial in regulating resistance to cancer treatment. In the future, targeting m6A modification and circRNAs in drug development may help researchers conquer treatment resistance.

## Data Availability

Data sharing not applicable to this article as no datasets were generated or analyzed during the current study.

## Abbreviations

circRNAs: Circular RNAs
CSCs: Cancer stem cells
ecRNAs: Exonic circRNAs
EIciRNAs: Exon-intron circRNAs
EMT: Epithelial-to-mesenchymal transition
HCC: Hepatocellular carcinoma
HSCC: Hypopharyngeal squamous cell carcinoma
IGF2BP3: Insulin-like growth factor 2 mRNA-binding protein 3
IRES: Internal ribosome entry site
m1A: N1-methyladenosine
m6A: N6-methyladenosine
m5C: 5-Methylcytosine
m7G: 7-Methylguanine
m6Am: 2'-O-dimethyladenosine
MeRIP-seq: Methylated RNA immunoprecipitation sequencing
m6A-label-seq: M6A labeling-based sequencing
miCLIP: Methylation-iCLIP
NSCLC: Non-small cell lung carcinoma
RBPs: RNA-binding proteins
TME: Tumor microenvironment
tricRNAs: TRNA intronic circRNAs

## References

[1] Boccaletto P, Machnicka MA, Purta E, Piatkowski P, Baginski B, Wirecki TK, de Crécy-Lagard V, Ross R, Limbach PA, Kotter A,: MODOMICS: a database of RNA modification pathways., et al. update. Nucleic Acids Res. 2017;2018(46):D303–d307. 10.1093/nar/gkx1030 PMC5753262 29106616

[2] Li J, Yang X, Qi Z, Sang Y, Liu Y, Xu B, Liu W, Xu Z, Deng Y. The role of mRNA m(6)A methylation in the nervous system. Cell Biosci. 2019;9:66. 31452869 10.1186/s13578-019-0330-y PMC6701067

[3] Wang X, Wu R, Liu Y, Zhao Y, Bi Z, Yao Y, Liu Q, Shi H, Wang F, Wang Y. m(6)A mRNA methylation controls autophagy and adipogenesis by targeting Atg5 and Atg7. Autophagy. 2020;16:1221–35. 31451060 10.1080/15548627.2019.1659617 PMC7469583

[4] Liang Z, Ye H, Ma J, Wei Z, Wang Y, Zhang Y, Huang D, Song B, Meng J, Rigden D, Chen K. m6A-Atlas v2.0: updated resources for unraveling the N6-methyladenosine (m6A) epitranscriptome among multiple species. Nucleic Acids Res. 2024;52(D1):D194-202. 10.1093/nar/gkad691. 10.1093/nar/gkad691 PMC10768109 37587690

[5] Jain S, Koziej L, Poulis P, Kaczmarczyk I, Gaik M, Rawski M, Ranjan N, Glatt S, Rodnina M. Modulation of translational decoding by mA modification of mRNA. Nat Commun. 2023;14:4784. 37553384 10.1038/s41467-023-40422-7 PMC10409866

[6] Su H, Cheung H, Lau H, Chen H, Zhang X, Qin N, Wang Y, Chan M, Wu W, Chen H: Crosstalk between gut microbiota and RNA N6-methyladenosine modification in cancer. FEMS Microbiol Rev. 2023;47(4):fuad036. 10.1093/femsre/fuad036. 10.1093/femsre/fuad036 37407433

[7] Liu Y, Yang D, Liu T, Chen J, Yu J, Yi P. N6-methyladenosine-mediated gene regulation and therapeutic implications. Trends Mol Med. 2023;29:454–67. 37068987 10.1016/j.molmed.2023.03.005

[8] He T, Zhang Q, Xu P, Tao W, Lin F, Liu R, Li M, Duan X, Cai C, Gu D, et al. Extracellular vesicle-circEHD2 promotes the progression of renal cell carcinoma by activating cancer-associated fibroblasts. Mol Cancer. 2023;22:117. 37481520 10.1186/s12943-023-01824-9 PMC10362694

[9] Circular RNA. detection pipelines yield divergent sets of circular RNAs. Nat Methods. 2023;20:1135–6. 37443339 10.1038/s41592-023-01945-5

[10] Liao Y, Qiu X, Liu J, Zhang Z, Liu B, Jin C: The role of m6A-modified CircEPHB4 in glioma pathogenesis: Insights into cancer stemness metastasis. Ann Clin Translat Neurol. 2023;10(10):1749-67. 10.1002/acn3.51864. 10.1002/acn3.51864 PMC10578901 37614011

[11] Li K, Peng Z, Wang R, Li X, Du N, Liu D, Zhang J, Zhang Y, Ma L, Sun Y, et al. Enhancement of TKI sensitivity in lung adenocarcinoma through m6A-dependent translational repression of Wnt signaling by circ-FBXW7. Mol Cancer. 2023;22:103. 37393311 10.1186/s12943-023-01811-0 PMC10314519

[12] Zhang F, Jiang J, Qian H, Yan Y, Xu W. Exosomal circRNA: emerging insights into cancer progression and clinical application potential. J Hematol Oncol. 2023;16:67. 37365670 10.1186/s13045-023-01452-2 PMC10294326

[13] Liu W, Tang T, Lu X, Fu X, Yang Y, Peng L: MPCLCDA: predicting circRNA-disease associations by using automatically selected meta-path and contrastive learning. Brief Bioinform. 2023;24(4):bbad227. 10.1093/bib/bbad227. 10.1093/bib/bbad227 37328701

[14] Wu P, Hou X, Peng M, Deng X, Yan Q, Fan C, Mo Y, Wang Y, Li Z, Wang F, et al. Circular RNA circRILPL1 promotes nasopharyngeal carcinoma malignant progression by activating the Hippo-YAP signaling pathway. Cell Death Differ. 2023;30:1679–94. 37173390 10.1038/s41418-023-01171-8 PMC10307875

[15] Ma C, Wang X, Zhang L, Zhu X, Bai J, He S, Mei J, Jiang J, Guan X, Zheng X, et al. Super enhancer-associated circular RNA-CircKrt4 regulates hypoxic pulmonary artery endothelial cell dysfunction in mice. Arterioscler Thromb Vasc Biol. 2023;43:1179–98. 37139839 10.1161/ATVBAHA.122.318842

[16] Fang Z, Mei W, Qu C, Lu J, Shang L, Cao F, Li F. Role of m6A writers, erasers and readers in cancer. Exp Hematol Oncol. 2022;11:45. 35945641 10.1186/s40164-022-00298-7 PMC9361621

[17] Liu T, Wei Q, Jin J, Luo Q, Liu Y, Yang Y, Cheng C, Li L, Pi J, Si Y, et al. The m6A reader YTHDF1 promotes ovarian cancer progression via augmenting EIF3C translation. Nucleic Acids Res. 2020;48:3816–31. 31996915 10.1093/nar/gkaa048 PMC7144925

[18] Liang J, Cai H, Hou C, Song F, Jiang Y, Wang Z, Qiu D, Zhu Y, Wang F, Yu D, Hou J: METTL14 inhibits malignant progression of oral squamous cell carcinoma by targeting the autophagy-related gene RB1CC1 in an m6A-IGF2BP2-dependent manner. Clin Sci. (London, England: 1979). 2023;137(17):1373-89. 10.1042/CS20230219. 10.1042/CS20230219 PMC10500204 37615536

[19] Qiu L, Jing Q, Li Y, Han J. RNA modification: mechanisms and therapeutic targets. Molecular biomedicine. 2023;4:25. 37612540 10.1186/s43556-023-00139-x PMC10447785

[20] Xiao W, Adhikari S, Dahal U, Chen YS, Hao YJ, Sun BF, Sun HY, Li A, Ping XL, Lai WY, et al. Nuclear m(6)A Reader YTHDC1 Regulates mRNA Splicing. Mol Cell. 2016;61:507–19. 26876937 10.1016/j.molcel.2016.01.012

[21] Ma C, Liao S, Zhu Z. Crystal structure of human YTHDC2 YTH domain. Biochem Biophys Res Commun. 2019;518:678–84. 31472957 10.1016/j.bbrc.2019.08.107

[22] Shi R, Ying S, Li Y, Zhu L, Wang X, Jin H. Linking the YTH domain to cancer: the importance of YTH family proteins in epigenetics. Cell Death Dis. 2021;12:346. 33795663 10.1038/s41419-021-03625-8 PMC8016981

[23] Scutenaire J, Deragon JM, Jean V, Benhamed M, Raynaud C, Favory JJ, Merret R, Bousquet-Antonelli C. The YTH Domain Protein ECT2 Is an m(6)A Reader Required for Normal Trichome Branching in Arabidopsis. Plant Cell. 2018;30:986–1005. 29618631 10.1105/tpc.17.00854 PMC6002185

[24] Yang Y, Hsu PJ, Chen YS, Yang YG. Dynamic transcriptomic m(6)A decoration: writers, erasers, readers and functions in RNA metabolism. Cell Res. 2018;28:616–24. 29789545 10.1038/s41422-018-0040-8 PMC5993786

[25] Huang Q, Mo J, Liao Z, Chen X, Zhang B. The RNA m(6)A writer WTAP in diseases: structure, roles, and mechanisms. Cell Death Dis. 2022;13:852. 36207306 10.1038/s41419-022-05268-9 PMC9546849

[26] Meyer KD, Jaffrey SR. Rethinking m(6)A Readers, Writers, and Erasers. Annu Rev Cell Dev Biol. 2017;33:319–42. 28759256 10.1146/annurev-cellbio-100616-060758 PMC5963928

[27] Flamand MN, Tegowski M, Meyer KD. The proteins of mrna modification: writers, readers, and erasers. Annu Rev Biochem. 2023;92:145–73. 37068770 10.1146/annurev-biochem-052521-035330 PMC10443600

[28] Fu Y, Jia G, Pang X, Wang RN, Wang X, Li CJ, Smemo S, Dai Q, Bailey KA, Nobrega MA, et al. FTO-mediated formation of N6-hydroxymethyladenosine and N6-formyladenosine in mammalian RNA. Nat Commun. 2013;4:1798. 23653210 10.1038/ncomms2822 PMC3658177

[29] Jia G, Yang CG, Yang S, Jian X, Yi C, Zhou Z, He C. Oxidative demethylation of 3-methylthymine and 3-methyluracil in single-stranded DNA and RNA by mouse and human FTO. FEBS Lett. 2008;582:3313–9. 18775698 10.1016/j.febslet.2008.08.019 PMC2577709

[30] Wu P, Fang X, Liu Y, Tang Y, Wang W, Li X, Fan Y. N6-methyladenosine modification of circCUX1 confers radioresistance of hypopharyngeal squamous cell carcinoma through caspase1 pathway. Cell Death Dis. 2021;12:298. 33741902 10.1038/s41419-021-03558-2 PMC7979824

[31] Chen Z, Pi H, Zheng W, Guo X, Shi C, Wang Z, Zhang J, Qu X, Liu L, Shen H, et al: The 3' Non-Coding Sequence Negatively Regulates PD-L1 Expression, and Its Regulators Are Systematically Identified in Pan-Cancer. Genes. 2023;14(8):1620. 10.3390/genes14081620. 10.3390/genes14081620 PMC10454350 37628671

[32] Deng X, Sun X, Hu Z, Wu Y, Zhou C, Sun J, Gao X, Huang Y. Exploring the role of m6A methylation regulators in glioblastoma multiforme and their impact on the tumor immune microenvironment. FASEB journal : official publication of the Federation of American Societies for Experimental Biology. 2023;37: e23155. 37606566 10.1096/fj.202301343

[33] Bai R, Sun M, Chen Y, Zhuo S, Song G, Wang T, Zhang Z. H19 recruited N 6 -methyladenosine (m 6 A) reader YTHDF1 to promote SCARB1 translation and facilitate angiogenesis in gastric cancer. Chin Med J. 2023;136:1719–31. 37279381 10.1097/CM9.0000000000002722 PMC10344490

[34] Chen RX, Chen X, Xia LP, Zhang JX, Pan ZZ, Ma XD, Han K, Chen JW, Judde JG, Deas O, et al. N(6)-methyladenosine modification of circNSUN2 facilitates cytoplasmic export and stabilizes HMGA2 to promote colorectal liver metastasis. Nat Commun. 2019;10:4695. 31619685 10.1038/s41467-019-12651-2 PMC6795808

[35] Chen C, Guo Y, Guo Y, Wu X, Si C, Xu Y, Kang Q, Sun Z. m6A Modification in Non-Coding RNA: The Role in Cancer Drug Resistance. Front Oncol. 2021;11: 746789. 34745970 10.3389/fonc.2021.746789 PMC8564146

[36] Huang J, Sun M, Tao Y, Ren J, Peng M, Jing Y, Xiao Q, Yang J, Lin C, Lei L, et al: Cytoplasmic Expression of TP53INP2 Modulated by Demethylase FTO and Mutant NPM1 Promotes Autophagy in Leukemia Cells. Int J Mole Sci. 2023;24(2):1624. 10.3390/ijms24021624. 10.3390/ijms24021624 PMC9865930 36675134

[37] Liu S, Guo X, Shang Q, Gao P. The biogenesis, biological functions and modification of Circular RNAs. Exp Mol Pathol. 2023;131:104861. 37156323 10.1016/j.yexmp.2023.104861

[38] Chen C, Yuan W, Zhou Q, Shao B, Guo Y, Wang W, Yang S, Guo Y, Zhao L, Dang Q, et al. N6-methyladenosine-induced circ1662 promotes metastasis of colorectal cancer by accelerating YAP1 nuclear localization. Theranostics. 2021;11:4298–315. 33754062 10.7150/thno.51342 PMC7977475

[39] Xu J, Wan Z, Tang M, Lin Z, Jiang S, Ji L, Gorshkov K, Mao Q, Xia S, Cen D, et al. N(6)-methyladenosine-modified CircRNA-SORE sustains sorafenib resistance in hepatocellular carcinoma by regulating β-catenin signaling. Mol Cancer. 2020;19:163. 33222692 10.1186/s12943-020-01281-8 PMC7681956

[40] Park OH, Ha H, Lee Y, Boo SH, Kwon DH, Song HK, Kim YK. Endoribonucleolytic Cleavage of m(6)A-Containing RNAs by RNase P/MRP Complex. Mol Cell. 2019;74:494–507.e498. 30930054 10.1016/j.molcel.2019.02.034

[41] Yang Y, Fan X, Mao M, Song X, Wu P, Zhang Y, Jin Y, Yang Y, Chen LL, Wang Y, et al. Extensive translation of circular RNAs driven by N(6)-methyladenosine. Cell Res. 2017;27:626–41. 28281539 10.1038/cr.2017.31 PMC5520850

[42] Chen YG, Chen R, Ahmad S, Verma R, Kasturi SP, Amaya L, Broughton JP, Kim J, Cadena C, Pulendran B, et al. N6-Methyladenosine Modification Controls Circular RNA Immunity. Mol Cell. 2019;76:96–109.e109. 31474572 10.1016/j.molcel.2019.07.016 PMC6778039

[43] Zhang L, Li Y, Zhou L, Zhou H, Ye L, Ou T, Hong H, Zheng S, Zhou Z, Wu K, et al. The m6A Reader YTHDF2 Promotes Bladder Cancer Progression by Suppressing RIG-I-Mediated Immune Response. Cancer Res. 2023;83:1834–50. 36939388 10.1158/0008-5472.CAN-22-2485 PMC10236158

[44] Jiang ZX, Wang YN, Li ZY, Dai ZH, He Y, Chu K, Gu JY, Ji YX, Sun NX, Yang F, Li W. The m6A mRNA demethylase FTO in granulosa cells retards FOS-dependent ovarian aging. Cell Death Dis. 2021;12:744. 34315853 10.1038/s41419-021-04016-9 PMC8316443

[45] Bao Y, Zhai J, Chen H, Wong CC, Liang C, Ding Y, Huang D, Gou H, Chen D, Pan Y, et al. Targeting m(6)A reader YTHDF1 augments antitumour immunity and boosts anti-PD-1 efficacy in colorectal cancer. Gut. 2023;72:1497–509. 36717220 10.1136/gutjnl-2022-328845 PMC10359538

[46] Weng H, Huang F, Yu Z, Chen Z, Prince E, Kang Y, Zhou K, Li W, Hu J, Fu C, et al. The m(6)A reader IGF2BP2 regulates glutamine metabolism and represents a therapeutic target in acute myeloid leukemia. Cancer Cell. 2022;40:1566–1582.e1510. 36306790 10.1016/j.ccell.2022.10.004 PMC9772162

[47] Ju G, Lei J, Cai S, Liu S, Yin X, Peng C: The Emerging, Multifaceted Role of WTAP in Cancer and Cancer Therapeutics. Cancers. 2023;15(11):3053. 10.3390/cancers15113053. 10.3390/cancers15113053 PMC10252703 37297015

[48] Sun X, Fu S, Yuan X, Pu X, Wang R, Wang X, Lu H: RNA N6-methyladenosine (m6A) modification in HNSCC: molecular mechanism and therapeutic potential. Cancer Gene Therapy. 2023;30(9):1209-14. 10.1038/s41417-023-00628-9. 10.1038/s41417-023-00628-9 37221404

[49] Wang L, Tang Y. N6-methyladenosine (m6A) in cancer stem cell: From molecular mechanisms to therapeutic implications. Biomed Pharmacother. 2023;163:114846. 37167725 10.1016/j.biopha.2023.114846

[50] Dong H, Zeng L, Chen W, Zhang Q, Wang F, Wu Y, Cui B, Qi J, Zhang X, Liu C, et al. N6-methyladenine-mediated aberrant activation of the lncRNA SOX2OT-GLI1 loop promotes non-small-cell lung cancer stemness. Cell death discovery. 2023;9:149. 37149646 10.1038/s41420-023-01442-w PMC10164154

[51] Zhang Z, Tan X, Wu R, Deng T, Wang H, Jiang X, Zeng P, Tang J. m6A-mediated upregulation of lncRNA-AC0263561 promotes cancer stem cell maintenance in lung adenocarcinoma via activating Wnt signaling pathway. Aging. 2023;15:3538–48. 37142269 10.18632/aging.204689 PMC10449284

[52] Chen J, Ye M, Bai J, Hu C, Lu F, Gu D, Yu P, Tang Q. el insights into the interplay between m6A modification and programmed cell death in cancer. Int J Biol Sci. 2023;19:1748–63. 37063421 10.7150/ijbs.81000 PMC10092764

[53] Zheng F, Xu R. CircPVT1 contributes to chemotherapy resistance of lung adenocarcinoma through miR-145-5p/ABCC1 axis. Biomed Pharmacother. 2020;124:109828. 31986409 10.1016/j.biopha.2020.109828

[54] He X, Ma J, Zhang M, Cui J, Yang H. Circ_0007031 enhances tumor progression and promotes 5-fluorouracil resistance in colorectal cancer through regulating miR-133b/ABCC5 axis. Cancer Biomark. 2020;29:531–42. 32865180 10.3233/CBM-200023 PMC12662547

[55] Xu QY, Xie MJ, Huang J, Wang ZW. Effect of circ MTHFD2 on resistance to pemetrexed in gastric cancer through regulating expression of miR-124. Eur Rev Med Pharmacol Sci. 2019;23:10290–9. 31841184 10.26355/eurrev_201912_19667

[56] Huang X, Li Z, Zhang Q, Wang W, Li B, Wang L, Xu Z, Zeng A, Zhang X, Zhang X, et al. Circular RNA AKT3 upregulates PIK3R1 to enhance cisplatin resistance in gastric cancer via miR-198 suppression. Mol Cancer. 2019;18:71. 30927924 10.1186/s12943-019-0969-3 PMC6441201

[57] Xu X, Zhang J, Tian Y, Gao Y, Dong X, Chen W, Yuan X, Yin W, Xu J, Chen K, et al. CircRNA inhibits DNA damage repair by interacting with host gene. Mol Cancer. 2020;19:128. 32838810 10.1186/s12943-020-01246-x PMC7446195

[58] Zhang Q, Miao Y, Fu Q, Hu H, Chen H, Zeng A, Jin Y, Jiang Y, Qian L, Wu L, et al. CircRNACCDC66 regulates cisplatin resistance in gastric cancer via the miR-618/BCL2 axis. Biochem Biophys Res Commun. 2020;526:713–20. 32253030 10.1016/j.bbrc.2020.03.156

[59] Liu Z, Gu S, Wu K, Li L, Dong C, Wang W, Zhou Y. CircRNA-DOPEY2 enhances the chemosensitivity of esophageal cancer cells by inhibiting CPEB4-mediated Mcl-1 translation. J Exp Clin Cancer Res. 2021;40:361. 34781999 10.1186/s13046-021-02149-5 PMC8591801

[60] Xu J, Ni L, Zhao F, Dai X, Tao J, Pan J, Shi A, Shen Z, Su C, Zhang Y. Overexpression of hsa_circ_0002874 promotes resistance of non-small cell lung cancer to paclitaxel by modulating miR-1273f/MDM2/p53 pathway. Aging (Albany NY). 2021;13:5986–6009. 33612481 10.18632/aging.202521 PMC7950269

[61] Xu G, Li M, Wu J, Qin C, Tao Y, He H. Circular RNA circNRIP1 Sponges microRNA-138-5p to Maintain Hypoxia-Induced Resistance to 5-Fluorouracil Through HIF-1α-Dependent Glucose Metabolism in Gastric Carcinoma. Cancer Manag Res. 2020;12:2789–802. 32425596 10.2147/CMAR.S246272 PMC7186590

[62] Zhang W, He Y, Zhang Y. CircRNA in ocular neovascular diseases: Fundamental mechanism and clinical potential. Pharmacol Res. 2023;197:106946. 37797661 10.1016/j.phrs.2023.106946

[63] Feng B, Chen K, Zhang W, Zheng Q, He Y. circPGAM1 enhances autophagy signaling during laryngocarcinoma drug resistance by regulating miR-376a. Biochem Biophys Res Commun. 2021;534:966–72. 33121682 10.1016/j.bbrc.2020.10.063

[64] Peng L, Sang H, Wei S, Li Y, Jin D, Zhu X, Li X, Dang Y, Zhang G. circCUL2 regulates gastric cancer malignant transformation and cisplatin resistance by modulating autophagy activation via miR-142-3p/ROCK2. Mol Cancer. 2020;19:156. 33153478 10.1186/s12943-020-01270-x PMC7643398

[65] Wang Q, Liang D, Shen P, Yu Y, Yan Y, You W. Hsa_circ_0092276 promotes doxorubicin resistance in breast cancer cells by regulating autophagy via miR-348/ATG7 axis. Transl Oncol. 2021;14: 101045. 34023560 10.1016/j.tranon.2021.101045 PMC8163983

[66] Yu S, Wang M, Zhang H, Guo X, Qin R: Circ_0092367 Inhibits EMT and Gemcitabine Resistance in Pancreatic Cancer via Regulating the miR-1206/ESRP1 Axis. Genes (Basel). 2021;12(11):1701. 10.3390/genes12111701. 10.3390/genes12111701 PMC8622583 34828307

[67] Chen C, Zhang M, Zhang Y. Circ_0000079 Decoys the RNA-Binding Protein FXR1 to Interrupt Formation of the FXR1/PRCKI Complex and Decline Their Mediated Cell Invasion and Drug Resistance in NSCLC. Cell Transplant. 2020;29:963689720961070. 32951448 10.1177/0963689720961070 PMC7784611

[68] Zhao Y, Zheng R, Chen J, Ning D. CircRNA CDR1as/miR-641/HOXA9 pathway regulated stemness contributes to cisplatin resistance in non-small cell lung cancer (NSCLC). Cancer Cell Int. 2020;20:289. 32655321 10.1186/s12935-020-01390-w PMC7339514

[69] Jian X, He H, Zhu J, Zhang Q, Zheng Z, Liang X, Chen L, Yang M, Peng K, Zhang Z, et al. Hsa_circ_001680 affects the proliferation and migration of CRC and mediates its chemoresistance by regulating BMI1 through miR-340. Mol Cancer. 2020;19:20. 32005118 10.1186/s12943-020-1134-8 PMC6993513

[70] Zhou S, Guo Z, Lv X, Zhang X. CircGOT1 promotes cell proliferation, mobility, and glycolysis-mediated cisplatin resistance via inhibiting its host gene GOT1 in esophageal squamous cell cancer. Cell Cycle. 2022;21:247–60. 34919012 10.1080/15384101.2021.2015671 PMC8855861

[71] Jiang X, Guo S, Wang S, Zhang Y, Chen H, Wang Y, Liu R, Niu Y, Xu Y. EIF4A3-Induced circARHGAP29 Promotes Aerobic Glycolysis in Docetaxel-Resistant Prostate Cancer through IGF2BP2/c-Myc/LDHA Signaling. Cancer Res. 2022;82:831–45. 34965937 10.1158/0008-5472.CAN-21-2988

[72] Tan WQ, Yuan L, Wu XY, He CG, Zhu SC, Ye M. Exosome-delivered circular RNA DLGAP4 induces chemoresistance via miR-143-HK2 axis in neuroblastoma. Cancer Biomark. 2022;34:375–84. 35068445 10.3233/CBM-210272 PMC12364187

[73] Shi Q, Ji T, Ma Z, Tan Q, Liang J: Serum Exosomes-Based Biomarker circ_0008928 Regulates Cisplatin Sensitivity, Tumor Progression, and Glycolysis Metabolism by miR-488/HK2 Axis in Cisplatin-Resistant Nonsmall Cell Lung Carcinoma. Cancer Biother Radiopharm 2021. 10.1089/cbr.2020.4490 33661058

[74] Yang W, Liu Y, Gao R, Xiu Z, Sun T. Knockdown of cZNF292 suppressed hypoxic human hepatoma SMMC7721 cell proliferation, vasculogenic mimicry, and radioresistance. Cell Signal. 2019;60:122–35. 31028816 10.1016/j.cellsig.2019.04.011

[75] Zhao Y, Zhong R, Deng C, Zhou Z. Circle RNA circABCB10 Modulates PFN2 to Promote Breast Cancer Progression, as Well as Aggravate Radioresistance Through Facilitating Glycolytic Metabolism Via miR-223-3p. Cancer Biother Radiopharm. 2021;36:477–90. 32522014 10.1089/cbr.2019.3389

[76] Huang Y, Dai Y, Wen C, He S, Shi J, Zhao D, Wu L, Zhou H. circSETD3 Contributes to Acquired Resistance to Gefitinib in Non-Small-Cell Lung Cancer by Targeting the miR-520h/ABCG2 Pathway. Mol Ther Nucleic Acids. 2020;21:885–99. 32805491 10.1016/j.omtn.2020.07.027 PMC7452060

[77] Cao HX, Miao CF, Sang LN, Huang YM, Zhang R, Sun L, Jiang ZX. Circ_0009910 promotes imatinib resistance through ULK1-induced autophagy by sponging miR-34a-5p in chronic myeloid leukemia. Life Sci. 2020;243: 117255. 31923418 10.1016/j.lfs.2020.117255

[78] Xu H, Chen R, Shen Q, Yang D, Peng H, Tong J, Fu Q. Overexpression of Circular RNA circ_0013587 Reverses Erlotinib Resistance in Pancreatic Cancer Cells Through Regulating the miR-1227/E-Cadherin Pathway. Front Oncol. 2021;11: 754146. 34552882 10.3389/fonc.2021.754146 PMC8450525

[79] Xu YP, Dong ZN, Wang SW, Zheng YM, Zhang C, Zhou YQ, Zhao YJ, Zhao Y, Wang F, Peng R, et al. circHMGCS1-016 reshapes immune environment by sponging miR-1236-3p to regulate CD73 and GAL-8 expression in intrahepatic cholangiocarcinoma. J Exp Clin Cancer Res. 2021;40:290. 34526098 10.1186/s13046-021-02095-2 PMC8442376

[80] Jia L, Wang Y, Wang CY. circFAT1 Promotes Cancer Stemness and Immune Evasion by Promoting STAT3 Activation. Adv Sci (Weinh). 2021;8:2003376. 34258151 10.1002/advs.202003376 PMC8261519

[81] Koppenol WH, Bounds PL, Dang CV. Otto Warburg’s contributions to current concepts of cancer metabolism. Nat Rev Cancer. 2011;11:325–37. 21508971 10.1038/nrc3038

[82] Ganapathy-Kanniappan S, Geschwind JF. Tumor glycolysis as a target for cancer therapy: progress and prospects. Mol Cancer. 2013;12:152. 24298908 10.1186/1476-4598-12-152 PMC4223729

[83] Marcucci F, Rumio C. Glycolysis-induced drug resistance in tumors-A response to danger signals? Neoplasia. 2021;23:234–45. 33418276 10.1016/j.neo.2020.12.009 PMC7804361

[84] Yu H, Yang X, Tang J, Si S, Zhou Z, Lu J, Han J, Yuan B, Wu Q, Lu Q, Yang H. ALKBH5 Inhibited Cell Proliferation and Sensitized Bladder Cancer Cells to Cisplatin by m6A-CK2α-Mediated Glycolysis. Mol Ther Nucleic Acids. 2021;23:27–41. 33376625 10.1016/j.omtn.2020.10.031 PMC7744648

[85] Tian Y, Xiao H, Yang Y, Zhang P, Yuan J, Zhang W, Chen L, Fan Y, Zhang J, Cheng H, et al. Crosstalk between 5-methylcytosine and N-methyladenosine machinery defines disease progression, therapeutic response and pharmacogenomic landscape in hepatocellular carcinoma. Mol Cancer. 2023;22:5. 36627693 10.1186/s12943-022-01706-6 PMC9830866

[86] Xu X, Zhang P, Huang Y, Shi W, Mao J, Ma N, Kong L, Guo L, Liu J, Chen J, Lu R. METTL3-mediated m6A mRNA contributes to the resistance of carbon-ion radiotherapy in non-small-cell lung cancer. Cancer Sci. 2023;114:105–14. 36114749 10.1111/cas.15590 PMC9807515

[87] Du H, Zou N, Zuo H, Zhang X, Zhu S. YTHDF3 mediates HNF1α regulation of cervical cancer radio-resistance by promoting RAD51D translation in an m6A-dependent manner. FEBS J. 2023;290:1920–35. 36380687 10.1111/febs.16681

[88] Li E, Xia M, Du Y, Long K, Ji F, Pan F, He L, Hu Z, Guo Z: METTL3 promotes homologous recombination repair and modulates chemotherapeutic response in breast cancer by regulating the EGF/RAD51 axis. eLife. 2022:11:e75231. 10.7554/eLife.75231. 10.7554/eLife.75231 PMC9094751 35502895

[89] Torii Y, Kato R, Minami Y, Hasegawa K, Fujii T, Udagawa Y. ERCC1 expression and chemosensitivity in uterine cervical adenocarcinoma cells. Anticancer Res. 2014;34:107–15. 24403450

[90] Li W, Jie Z, Li Z, Liu Y, Gan Q, Mao Y, Wang X. ERCC1 siRNA ameliorates drug resistance to cisplatin in gastric carcinoma cell lines. Mol Med Rep. 2014;9:2423–8. 24699918 10.3892/mmr.2014.2112

[91] Zhou S, Bai ZL, Xia D, Zhao ZJ, Zhao R, Wang YY, Zhe H. FTO regulates the chemo-radiotherapy resistance of cervical squamous cell carcinoma (CSCC) by targeting β-catenin through mRNA demethylation. Mol Carcinog. 2018;57:590–7. 29315835 10.1002/mc.22782

[92] Wright WD, Shah SS, Heyer WD. Homologous recombination and the repair of DNA double-strand breaks. J Biol Chem. 2018;293:10524–35. 29599286 10.1074/jbc.TM118.000372 PMC6036207

[93] Zhou K, Sun Y, Dong D, Zhao C, Wang W. EMP3 negatively modulates breast cancer cell DNA replication, DNA damage repair, and stem-like properties. Cell Death Dis. 2021;12:844. 34511602 10.1038/s41419-021-04140-6 PMC8435533

[94] Wei J, Yin Y, Zhou J, Chen H, Peng J, Yang J, Tang Y. METTL3 potentiates resistance to cisplatin through m(6) A modification of TFAP2C in seminoma. J Cell Mol Med. 2020;24:11366–80. 32857912 10.1111/jcmm.15738 PMC7576266

[95] Zhou M, Liu W, Zhang J, Sun N. RNA mA Modification in Immunocytes and DNA Repair: The Biological Functions and Prospects in Clinical Application. Frontiers in cell and developmental biology. 2021;9: 794754. 34988083 10.3389/fcell.2021.794754 PMC8722703

[96] Huang C, Zhou S, Zhang C, Jin Y, Xu G, Zhou L, Ding G, Pang T, Jia S, Cao L. ZC3H13-mediated N6-methyladenosine modification of PHF10 is impaired by fisetin which inhibits the DNA damage response in pancreatic cancer. Cancer Lett. 2022;530:16–28. 35033590 10.1016/j.canlet.2022.01.013

[97] Li S, Li N, Li B, Zhu L, Xu T, Wang L, Zhang J, Kong F: CircHIPK3 promotes proliferation and metastasis of villous trophoblasts through miR-30a-3p/Wnt2 axis. J Genet. 2022;101:55. 36560845

[98] Ren S, Liu J, Feng Y, Li Z, He L, Li L, Cao X, Wang Z, Zhang Y. Knockdown of circDENND4C inhibits glycolysis, migration and invasion by up-regulating miR-200b/c in breast cancer under hypoxia. J Exp Clin Cancer Res. 2019;38:388. 31488193 10.1186/s13046-019-1398-2 PMC6727545

[99] Hayashi M, Sugahara K, Yamamura A, Iida Y. Evaluation of the Properties of the DNA Methyltransferase from Aeropyrum pernix K1. Microbiology spectrum. 2021;9:e0018621. 34585946 10.1128/Spectrum.00186-21 PMC8557920

[100] Miranda-Gonçalves V, Lobo J, Guimarães-Teixeira C, Barros-Silva D, Guimarães R, Cantante M, Braga I, Maurício J, Oing C, Honecker F, et al. The component of the mA writer complex VIRMA is implicated in aggressive tumor phenotype, DNA damage response and cisplatin resistance in germ cell tumors. Journal of experimental & clinical cancer research : CR. 2021;40:268. 34446080 10.1186/s13046-021-02072-9 PMC8390281

[101] Yu F, Wei J, Cui X, Yu C, Ni W, Bungert J, Wu L, He C, Qian Z. Post-translational modification of RNA m6A demethylase ALKBH5 regulates ROS-induced DNA damage response. Nucleic Acids Res. 2021;49:5779–97. 34048572 10.1093/nar/gkab415 PMC8191756

[102] Qu F, Tsegay P, Liu Y. N-Methyladenosine, DNA Repair, and Genome Stability. Front Mol Biosci. 2021;8:645823. 33898522 10.3389/fmolb.2021.645823 PMC8062805

[103] Li R, Chen H, Li C, Qi Y, Zhao K, Wang J, You C, Huang H. The prognostic value and immune landscaps of m6A/m5C-related lncRNAs signature in the low grade glioma. BMC Bioinformatics. 2023;24:274. 37403043 10.1186/s12859-023-05386-x PMC10320943

[104] Wang L, Peng J. METTL5 serves as a diagnostic and prognostic biomarker in hepatocellular carcinoma by influencing the immune microenvironment. Sci Rep. 2023;13:10755. 37400463 10.1038/s41598-023-37807-5 PMC10318095

[105] Huang Y, Guan Y, Zhang X: METTL3-Mediated Maturation of miR-99a-5p Promotes Cell Migration and Invasion in Oral Squamous Cell Carcinoma by Targeting ZBTB7A. Mol Biotechnol. 2023 Jul 27. 10.1007/s12033-023-00815-x. 10.1007/s12033-023-00815-x 37498409

[106] Hu B, Gao J, Shi J, Wen P, Guo W, Zhang S: m6A reader YTHDF3 triggers the progression of hepatocellular carcinoma through the YTHDF3/m A-EGFR/STAT3 axis and EMT. Mole Carcinogenesis 2023;62(10):1599-1614. 10.1002/mc.23602. 10.1002/mc.23602 37449789

[107] Shen S, Jin H, Zhang X, Zhang Y, Li X, Yan W, Xie S, Yu B, Hu J, Liu H, et al. LINC00426, a novel mA-regulated long non-coding RNA, induces EMT in cervical cancer by binding to ZEB1. Cell Signal. 2023;109:110788. 37392859 10.1016/j.cellsig.2023.110788

[108] Chen M, Tian B, Hu G, Guo Y: circUHRF2METTL3-Modulated Promotes Colorectal Cancer Stemness and Metastasis through Increasing mRNA Stability by Recruiting IGF2BP1. Cancers. 2023;15(12):3148. 10.3390/cancers15123148. 10.3390/cancers15123148 PMC10295973 37370759

[109] Xi S, Ming D, Zhang J, Guo M, Wang S, Cai Y, Liu M, Wang D, Zhang Y, Li Y, Yuan S. Downregulation of N6-methyladenosine-modified LINC00641 promotes EMT, but provides a ferroptotic vulnerability in lung cancer. Cell Death Dis. 2023;14:359. 37311754 10.1038/s41419-023-05880-3 PMC10264399

[110] Yang X, Bai Q, Chen W, Liang J, Wang F, Gu W, Liu L, Li Q, Chen Z, Zhou A, et al: m A-Dependent Modulation via IGF2BP3/MCM5/Notch Axis Promotes Partial EMT and LUAD Metastasis. Advanced science (Weinheim, Baden-Wurttemberg, Germany) 2023;10:e2206744. 10.1002/advs.202206744 PMC10369244 37171793

[111] Zhang Y, Wang L, Yan F, Yang M, Gao H, Zeng Y. Mettl3 Mediated m6A Methylation Involved in Epithelial-Mesenchymal Transition by Targeting SOCS3/STAT3/SNAI1 in Cigarette Smoking-Induced COPD. Int J Chron Obstruct Pulmon Dis. 2023;18:1007–17. 37275442 10.2147/COPD.S398289 PMC10239240

[112] Chen H, Zhang J, Yang L, Li Y, Wang Z, Ye C. circ-ZEB1 regulates epithelial-mesenchymal transition and chemotherapy resistance of colorectal cancer through acting on miR-200c-5p. Transl Oncol. 2023;28: 101604. 36542990 10.1016/j.tranon.2022.101604 PMC9792398

[113] Liu X, Su K, Sun X, Jiang Y, Wang L, Hu C, Zhang C, Lu M, Du X, Xing B. Sec62 promotes stemness and chemoresistance of human colorectal cancer through activating Wnt/β-catenin pathway. J Exp Clin Cancer Res. 2021;40:132. 33858476 10.1186/s13046-021-01934-6 PMC8051072

[114] Hu W, Klümper N, Schmidt D, Ritter M, Ellinger J, Hauser S. Depletion of the mA demethylases FTO and ALKBH5 impairs growth and metastatic capacity through EMT phenotype change in clear cell renal cell carcinoma. American journal of translational research. 2023;15:1744–55. 37056835 PMC10086911

[115] Wang Y, Chen Y, Liang J, Jiang M, Zhang T, Wan X, Wu J, Li X, Chen J, Sun J, et al: METTL3-mediated m6A modification of HMGA2 mRNA promotes subretinal fibrosis and epithelial-mesenchymal transition. J Mole Cell Biol. 2023;15(3):mjad005. 10.1093/jmcb/mjad005. 10.1093/jmcb/mjad005 PMC10603769 36945110

[116] Wang C. Danli Ma, Yu H, Zhuo Z, Ye Z: N6-methyladenosine (m6A) as a regulator of carcinogenesis and drug resistance by targeting epithelial-mesenchymal transition and cancer stem cells. Heliyon. 2023;9: e14001. 36915498 10.1016/j.heliyon.2023.e14001 PMC10006539

[117] Liang Y, Wang N, Zhang Y, Jiang W, Fang C, Feng Y, Ma H, Jiang F, Dong G. Self-restricted circular RNA circSOX2 suppressed the malignant progression in SOX2-amplified LUSC. Cell Death Dis. 2022;13:873. 36243874 10.1038/s41419-022-05288-5 PMC9568965

[118] Zhao S, Xu F, Ji Y, Wang Y, Wei M, Zhang L. Circular RNA circ-CD44 regulates chemotherapy resistance by targeting the miR-330-5p/ABCC1 axis in colorectal cancer cells. Histol Histopathol. 2023;38:209–21. 36106650 10.14670/HH-18-516

[119] Zhao F, Zhao P, Chang J, Sun X, Ma X, Shi B, Yin M, Wang Y, Yang Y. Identification and vitro verification of the potential drug targets of active ingredients of Chonglou in the treatment of lung adenocarcinoma based on EMT-related genes. Front Genet. 2023;14:1112671. 36824434 10.3389/fgene.2023.1112671 PMC9942681

[120] Chen C, Huang J, Huang J, Deng J, Shangguan X, Chen A, Chen L, Wu W. Metformin attenuates multiple myeloma cell proliferation and encourages apoptosis by suppressing METTL3-mediated m6A methylation of THRAP3, RBM25, and USP4. Cell cycle (Georgetown, Tex). 2023;22:986–1004. 36762777 10.1080/15384101.2023.2170521 PMC10054227

[121] Duan Y, Du Y, Mu Y, Gu Z, Wang C. Prognostic value, immune signature and molecular mechanisms of the SUMO family in pancreatic adenocarcinoma. Front Mol Biosci. 2022;9:1096679. 36589239 10.3389/fmolb.2022.1096679 PMC9798011

[122] Garg R, Melstrom L, Chen J, He C, Goel A: Targeting FTO Suppresses Pancreatic Carcinogenesis via Regulating Stem Cell Maintenance and EMT Pathway. Cancers. 2022;14(23):5919. 10.3390/cancers14235919. 10.3390/cancers14235919 PMC9737034 36497402

[123] Li Q, Ni Y, Zhang L, Jiang R, Xu J, Yang H, Hu Y, Qiu J, Pu L, Tang J, Wang X. HIF-1α-induced expression of m6A reader YTHDF1 drives hypoxia-induced autophagy and malignancy of hepatocellular carcinoma by promoting ATG2A and ATG14 translation. Signal Transduct Target Ther. 2021;6:76. 33619246 10.1038/s41392-020-00453-8 PMC7900110

[124] Feng S, Qiu G, Yang L, Feng L, Fan X, Ren F, Huang K, Chen Y: Omeprazole improves chemosensitivity of gastric cancer cells by m6A demethylase FTO-mediated activation of mTORC1 and DDIT3 up-regulation. Biosci Rep. 2021;41(1):BSR20200842. 10.1042/BSR20200842. 10.1042/BSR20200842 PMC7843496 33393595

[125] Chen H, Xiang Y, Yin Y, Peng J, Peng D, Li D, Kitazawa R, Tang Y, Yang J. The m6A methyltransferase METTL3 regulates autophagy and sensitivity to cisplatin by targeting ATG5 in seminoma. Transl Androl Urol. 2021;10:1711–22. 33968659 10.21037/tau-20-1411 PMC8100844

[126] Li J, Cao H, Yang J, Wang B. CircCDK1 blocking IGF2BP2-mediated m6A modification of CPPED1 promotes laryngeal squamous cell carcinoma metastasis via the PI3K/AKT signal pathway. Gene. 2023;884:147686. 37543219 10.1016/j.gene.2023.147686

[127] Chen X, Zhu S, Li H, Wang J, Sun L, Xu J, Hui Y, Li X, Li L, Zhao Y, et al. N-methyladenosine-modified circIRF2, identified by YTHDF2, suppresses liver fibrosis via facilitating FOXO3 nuclear translocation. Int J Biol Macromol. 2023;248:125811 37467831 10.1016/j.ijbiomac.2023.125811

[128] Qi K, Dou Y, Zhang Z, Wei Y, Song C, Qiao R, Li X, Yang F, Wang K, Li X, Han X: Expression Profile and Regulatory Properties of m6A-Modified circRNAs in the Longissimus Dorsi of Queshan Black and Large White Pigs. Animals. 2023;13(13):2190. 10.3390/ani13132190. 10.3390/ani13132190 PMC10339870 37443988

[129] Lin Z, Li J, Zhang J, Feng W, Lu J, Ma X, Ding W, Ouyang S, Lu J, Yue P, et al. Metabolic Reprogramming Driven by IGF2BP3 Promotes Acquired Resistance to EGFR Inhibitors in Non-Small Cell Lung Cancer. Can Res. 2023;83:2187–207. 10.1158/0008-5472.CAN-22-3059 37061993

[130] Yang L, Yan B, Qu L, Ren J, Li Q, Wang J, Kan X, Liu M, Wang Y, Sun Y, et al. IGF2BP3 Regulates TMA7-mediated Autophagy and Cisplatin Resistance in Laryngeal Cancer via m6A RNA Methylation. Int J Biol Sci. 2023;19:1382–400. 37056932 10.7150/ijbs.80921 PMC10086756

[131] Sun Y, Shen W, Hu S, Lyu Q, Wang Q, Wei T, Zhu W, Zhang J. METTL3 promotes chemoresistance in small cell lung cancer by inducing mitophagy. Journal of experimental & clinical cancer research : CR. 2023;42:65. 36932427 10.1186/s13046-023-02638-9 PMC10022264

[132] Huang H, Weng H, Sun W, Qin X, Shi H, Wu H, Zhao BS, Mesquita A, Liu C, Yuan CL, et al. Recognition of RNA N(6)-methyladenosine by IGF2BP proteins enhances mRNA stability and translation. Nat Cell Biol. 2018;20:285–95. 29476152 10.1038/s41556-018-0045-z PMC5826585

[133] Yang Z, Zhao F, Gu X, Feng L, Xu M, Li T, Liu X, Zhang X. Binding of RNA m6A by IGF2BP3 triggers chemoresistance of HCT8 cells via upregulation of ABCB1. Am J Cancer Res. 2021;11:1428–45. 33948366 PMC8085870

[134] Liu X, Yuan J, Zhang X, Li L, Dai X, Chen Q, Wang Y. ATF3 Modulates the Resistance of Breast Cancer Cells to Tamoxifen through an N(6)-Methyladenosine-Based Epitranscriptomic Mechanism. Chem Res Toxicol. 2021;34:1814–21. 34213887 10.1021/acs.chemrestox.1c00206 PMC8756675

[135] Hao CC, Xu CY, Zhao XY, Luo JN, Wang G, Zhao LH, Ge X, Ge XF. Up-regulation of VANGL1 by IGF2BPs and miR-29b-3p attenuates the detrimental effect of irradiation on lung adenocarcinoma. J Exp Clin Cancer Res. 2020;39:256. 33228740 10.1186/s13046-020-01772-y PMC7687693

[136] Liu Z, Wu K, Gu S, Wang W, Xie S, Lu T, Li L, Dong C, Wang X, Zhou Y. A methyltransferase-like 14/miR-99a-5p/tribble 2 positive feedback circuit promotes cancer stem cell persistence and radioresistance via histone deacetylase 2-mediated epigenetic modulation in esophageal squamous cell carcinoma. Clin Transl Med. 2021;11: e545. 34586732 10.1002/ctm2.545 PMC8441142

[137] Zhao H, Huang Y, Shi J, Dai Y, Wu L, Zhou H. ABCC10 Plays a Significant Role in the Transport of Gefitinib and Contributes to Acquired Resistance to Gefitinib in NSCLC. Front Pharmacol. 2018;9:1312. 30515095 10.3389/fphar.2018.01312 PMC6256088

[138] Xiao P, Liu YK, Han W, Hu Y, Zhang BY, Liu WL. Exosomal Delivery of FTO Confers Gefitinib Resistance to Recipient Cells through ABCC10 Regulation in an m6A-dependent Manner. Mol Cancer Res. 2021;19:726–38. 33563765 10.1158/1541-7786.MCR-20-0541

[139] Fulda S. Tumor resistance to apoptosis. Int J Cancer. 2009;124:511–5. 19003982 10.1002/ijc.24064

[140] Villanova L, Careccia S, De Maria R, Fiori ME: Micro-Economics of Apoptosis in Cancer: ncRNAs Modulation of BCL-2 Family Members. Int J Mol Sci. 2018;19(4):958. 10.3390/ijms19040958. 10.3390/ijms19040958 PMC5979352 29570632

[141] Wang H, Xu B, Shi J. N6-methyladenosine METTL3 promotes the breast cancer progression via targeting Bcl-2. Gene. 2020;722: 144076. 31454538 10.1016/j.gene.2019.144076

[142] Gu M, Zheng W, Zhang M, Dong X, Zhao Y, Wang S, Jiang H, Zheng X. LncRNA NONHSAT141924 promotes paclitaxel chemotherapy resistance through p-CREB/Bcl-2 apoptosis signaling pathway in breast cancer. J Cancer. 2020;11:3645–54. 32284761 10.7150/jca.39463 PMC7150466

[143] Cittelly DM, Das PM, Salvo VA, Fonseca JP, Burow ME, Jones FE. Oncogenic HER2{Delta}16 suppresses miR-15a/16 and deregulates BCL-2 to promote endocrine resistance of breast tumors. Carcinogenesis. 2010;31:2049–57. 20876285 10.1093/carcin/bgq192 PMC2994280

[144] Kalliokoski A, Niemi M. Impact of OATP transporters on pharmacokinetics. Br J Pharmacol. 2009;158:693–705. 19785645 10.1111/j.1476-5381.2009.00430.x PMC2765590

[145] Zhou T, Li S, Xiang D, Liu J, Sun W, Cui X, Ning B, Li X, Cheng Z, Jiang W, et al. m6A RNA methylation-mediated HNF3γ reduction renders hepatocellular carcinoma dedifferentiation and sorafenib resistance. Signal Transduct Target Ther. 2020;5:296. 33361765 10.1038/s41392-020-00299-0 PMC7762754

[146] An Y, Duan H. The role of m6A RNA methylation in cancer metabolism. Mol Cancer. 2022;21:14. 35022030 10.1186/s12943-022-01500-4 PMC8753874

[147] Zhu Y, Tan J, Goon J: Cuproptosis- and m6A-Related lncRNAs for Prognosis of Hepatocellular Carcinoma. Biology. 2023;12(8):1101. 10.3390/biology12081101. 10.3390/biology12081101 PMC10451969 37626987

[148] Yuan F, Cai X, Wang Y, Du C, Cong Z, Zeng X, Tang C, Ma C. Comprehensive analysis of mA subtype classification for immune microenvironment of pituitary adenomas. Int Immunopharmacol. 2023;124: 110784. 37607464 10.1016/j.intimp.2023.110784

[149] Ma C, Zheng Q, Wang Y, Li G, Zhao M, Sun Z: Pan-cancer analysis and experimental validation revealed the m6A methyltransferase KIAA1429 as a potential biomarker for diagnosis, prognosis, and immunotherapy. Aging. 2023;15(17):8664-8691. 10.18632/aging.204968. 10.18632/aging.204968 PMC10522386 37606975

[150] Yue S, Liu H, Su H, Luo C, Liang H, Zhang B, Zhang W. m6A-regulated tumor glycolysis: new advances in epigenetics and metabolism. Mol Cancer. 2023;22:137. 37582735 10.1186/s12943-023-01841-8 PMC10426175

[151] Xu W, Li H, Hameed Y, Abdel-Maksoud M, Almutairi S, Mubarak A, Aufy M, Alturaiki W, Alshalani A, Mahmoud A, Li C. Elucidating the clinical and immunological value of m6A regulator-mediated methylation modification patterns in adrenocortical carcinoma. Oncol Res. 2023;31:819–31. 37547754 10.32604/or.2023.029414 PMC10398396

[152] Lian B, Yan S, Li J, Bai Z, Li J. HNRNPC promotes collagen fiber alignment and immune evasion in breast cancer via activation of the VIRMA-mediated TFAP2A/DDR1 axis. Molecular medicine (Cambridge, Mass). 2023;29:103. 37528369 10.1186/s10020-023-00696-5 PMC10394847

[153] Xiao Y, Li J, Wu J. Development and validation of a novel prognostic signature based on m6A/m5C/m1A-related genes in hepatocellular carcinoma. BMC Med Genomics. 2023;16:177. 37525171 10.1186/s12920-023-01611-x PMC10391842

[154] Zhang Z, Gao W, Liu Z, Yu S, Jian H, Hou Z, Zeng P. Comprehensive analysis of m6A regulators associated with immune infiltration in Hepatitis B virus-related hepatocellular carcinoma. BMC Gastroenterol. 2023;23:259. 37507670 10.1186/s12876-023-02873-6 PMC10385918

[155] Hu J, Xue C, Wang Q. N-methyladenosine modification: an important player in the tumor immune microenvironment. Biomed Pharmacother. 2023;165:15171. 10.1016/j.biopha.2023.115171 37494788

[156] Najafi M, Mortezaee K, Majidpoor J. Cancer stem cell (CSC) resistance drivers. Life Sci. 2019;234:116781. 31430455 10.1016/j.lfs.2019.116781

[157] Khan RIN, Malla WA. m(6)A modification of RNA and its role in cancer, with a special focus on lung cancer. Genomics. 2021;113:2860–9. 34118382 10.1016/j.ygeno.2021.06.013

[158] Liu S, Jiang Z, Xiao P, Li X, Chen Y, Tang H, Chai Y, Liu Y, Zhu Z, Xie Q, et al. Hsa_circ_0005576 promotes osimertinib resistance through the miR-512-5p/IGF1R axis in lung adenocarcinoma cells. Cancer Sci. 2022;113:79–90. 34706132 10.1111/cas.15177 PMC8748248

[159] Lei M, Zheng G, Ning Q, Zheng J, Dong D. Translation and functional roles of circular RNAs in human cancer. Mol Cancer. 2020;19:30. 32059672 10.1186/s12943-020-1135-7 PMC7023758

[160] Li C, Li W, Cao S, Xu J, Qian Y, Pan X, Lei D, Wei D. Circ_0058106 promotes proliferation, metastasis and EMT process by regulating Wnt2b/β-catenin/c-Myc pathway through miR-185-3p in hypopharyngeal squamous cell carcinoma. Cell Death Dis. 2021;12:1063. 34750351 10.1038/s41419-021-04346-8 PMC8575998

[161] Wang Z, Wei P, Wei D, Cao S, Liu H, Chen L, Han X, Zhao X, Liu C, Li G, et al. Effect of up-regulation of circMATR3 on the proliferation, metastasis, progression and survival of hypopharyngeal carcinoma. J Cell Mol Med. 2020;24:4687–97. 32166857 10.1111/jcmm.15134 PMC7176838

[162] Rao X, Lai L, Li X, Wang L, Li A, Yang Q. N(6) -methyladenosine modification of circular RNA circ-ARL3 facilitates Hepatitis B virus-associated hepatocellular carcinoma via sponging miR-1305. IUBMB Life. 2021;73:408–17. 33372396 10.1002/iub.2438

[163] Qin S, Mao Y, Chen X, Xiao J, Qin Y, Zhao L. The functional roles, cross-talk and clinical implications of m6A modification and circRNA in hepatocellular carcinoma. Int J Biol Sci. 2021;17:3059–79. 34421350 10.7150/ijbs.62767 PMC8375232

[164] Rong D, Wu F, Lu C, Sun G, Shi X, Chen X, Dai Y, Zhong W, Hao X, Zhou J, et al. m6A modification of circHPS5 and hepatocellular carcinoma progression through HMGA2 expression. Mol Ther Nucleic Acids. 2021;26:637–48. 34703649 10.1016/j.omtn.2021.09.001 PMC8517093

[165] Xu J, Ji L, Liang Y, Wan Z, Zheng W, Song X, Gorshkov K, Sun Q, Lin H, Zheng X, et al. CircRNA-SORE mediates sorafenib resistance in hepatocellular carcinoma by stabilizing YBX1. Signal Transduct Target Ther. 2020;5:298. 33361760 10.1038/s41392-020-00375-5 PMC7762756

[166] Liu Z, Wang T, She Y, Wu K, Gu S, Li L, Dong C, Chen C, Zhou Y. N(6)-methyladenosine-modified circIGF2BP3 inhibits CD8(+) T-cell responses to facilitate tumor immune evasion by promoting the deubiquitination of PD-L1 in non-small cell lung cancer. Mol Cancer. 2021;20:105. 34416901 10.1186/s12943-021-01398-4 PMC8377850

[167] Tobiume K, Matsuzawa A, Takahashi T, Nishitoh H, Morita K, Takeda K, Minowa O, Miyazono K, Noda T, Ichijo H. ASK1 is required for sustained activations of JNK/p38 MAP kinases and apoptosis. EMBO Rep. 2001;2:222–8. 11266364 10.1093/embo-reports/kve046 PMC1083842

[168] Chen X, Ma W, Yao Y, Zhang Q, Li J, Wu X, Mei C, Jiang X, Chen Y, Wang G, et al. Serum deprivation-response protein induces apoptosis in hepatocellular carcinoma through ASK1-JNK/p38 MAPK pathways. Cell Death Dis. 2021;12:425. 33931585 10.1038/s41419-021-03711-x PMC8087765

[169] Wang T, Liu Z, She Y, Deng J, Zhong Y, Zhao M, Li S, Xie D, Sun X, Hu X, Chen C. A novel protein encoded by circASK1 ameliorates gefitinib resistance in lung adenocarcinoma by competitively activating ASK1-dependent apoptosis. Cancer Lett. 2021;520:321–31. 34389432 10.1016/j.canlet.2021.08.007

[170] Li B, Zhu L, Lu C, Wang C, Wang H, Jin H, Ma X, Cheng Z, Yu C, Wang S, et al. circNDUFB2 inhibits non-small cell lung cancer progression via destabilizing IGF2BPs and activating anti-tumor immunity. Nat Commun. 2021;12:295. 33436560 10.1038/s41467-020-20527-z PMC7804955

[171] Chen S, Shen X. Long noncoding RNAs: functions and mechanisms in colon cancer. Mol Cancer. 2020;19:167. 33246471 10.1186/s12943-020-01287-2 PMC7697375

[172] Cai J, Chen Z, Zhang Y, Wang J, Zhang Z, Wu J, Mao J, Zuo X. CircRHBDD1 augments metabolic rewiring and restricts immunotherapy efficacy via m(6)A modification in hepatocellular carcinoma. Mol Ther Oncolytics. 2022;24:755–71. 35317519 10.1016/j.omto.2022.02.021 PMC8908059

[173] Wei W, Sun J, Zhang H, Xiao X, Huang C, Wang L, Zhong H, Jiang Y, Zhang X, Jiang G. Circ0008399 Interaction with WTAP Promotes Assembly and Activity of the m(6)A Methyltransferase Complex and Promotes Cisplatin Resistance in Bladder Cancer. Cancer Res. 2021;81:6142–56. 34702726 10.1158/0008-5472.CAN-21-1518

[174] Sun K, Du Y, Hou Y, Zhao M, Li J, Du Y, Zhang L, Chen C, Yang H, Yan F, Su R. Saikosaponin D exhibits anti-leukemic activity by targeting FTO/m(6)A signaling. Theranostics. 2021;11:5831–46. 33897884 10.7150/thno.55574 PMC8058711

[175] Koukourakis MI, Giatromanolaki A, Panteliadou M, Pouliliou SE, Chondrou PS, Mavropoulou S, Sivridis E. Lactate dehydrogenase 5 isoenzyme overexpression defines resistance of prostate cancer to radiotherapy. Br J Cancer. 2014;110:2217–23. 24714743 10.1038/bjc.2014.158 PMC4007238

[176] Jeck WR, Sharpless NE. Detecting and characterizing circular RNAs. Nat Biotechnol. 2014;32:453–61. 24811520 10.1038/nbt.2890 PMC4121655

[177] Sulaiman SA, Abdul Murad NA, Mohamad Hanif EA, Abu N, Jamal R. Prospective Advances in Circular RNA Investigation. Adv Exp Med Biol. 2018;1087:357–70. 30259380 10.1007/978-981-13-1426-1_28

[178] Saenz-Pipaon G, San Martín P, Planell N, Maillo A, Ravassa S, Vilas-Zornoza A, Martinez-Aguilar E, Rodriguez JA, Alameda D, Lara-Astiaso D, et al. Functional and transcriptomic analysis of extracellular vesicles identifies calprotectin as a new prognostic marker in peripheral arterial disease (PAD). J Extracell Vesicles. 2020;9:1729646. 32158521 10.1080/20013078.2020.1729646 PMC7048174

[179] Wen G, Zhou T, Gu W. The potential of using blood circular RNA as liquid biopsy biomarker for human diseases. Protein Cell. 2021;12:911–46. 33131025 10.1007/s13238-020-00799-3 PMC8674396

[180] Wang Y, Jia G. Detection methods of epitranscriptomic mark N6-methyladenosine. Essays Biochem. 2020;64:967–79. 33284953 10.1042/EBC20200039

[181] Meyer KD, Saletore Y, Zumbo P, Elemento O, Mason CE, Jaffrey SR. Comprehensive analysis of mRNA methylation reveals enrichment in 3’ UTRs and near stop codons. Cell. 2012;149:1635–46. 22608085 10.1016/j.cell.2012.05.003 PMC3383396

[182] Shu X, Cao J, Cheng M, Xiang S, Gao M, Li T, Ying X, Wang F, Yue Y, Lu Z, et al. A metabolic labeling method detects m(6)A transcriptome-wide at single base resolution. Nat Chem Biol. 2020;16:887–95. 32341503 10.1038/s41589-020-0526-9

[183] Werner S, Galliot A, Pichot F, Kemmer T, Marchand V, Sednev MV, Lence T, Roignant JY, König J, Höbartner C, et al. NOseq: amplicon sequencing evaluation method for RNA m6A sites after chemical deamination. Nucleic Acids Res. 2021;49:e23. 33313868 10.1093/nar/gkaa1173 PMC7913672

[184] Nombela P, Miguel-López B, Blanco S. The role of m(6)A, m(5)C and Ψ RNA modifications in cancer: Novel therapeutic opportunities. Mol Cancer. 2021;20:18. 33461542 10.1186/s12943-020-01263-w PMC7812662

[185] Deng LJ, Deng WQ, Fan SR, Chen MF, Qi M, Lyu WY, Qi Q, Tiwari AK, Chen JX, Zhang DM, Chen ZS. m6A modification: recent advances, anticancer targeted drug discovery and beyond. Mol Cancer. 2022;21:52. 35164788 10.1186/s12943-022-01510-2 PMC8842557

[186] Wang Y, Liu J, Ma J, Sun T, Zhou Q, Wang W, Wang G, Wu P, Wang H, Jiang L, et al. Exosomal circRNAs: biogenesis, effect and application in human diseases. Mol Cancer. 2019;18:116. 31277663 10.1186/s12943-019-1041-z PMC6610963

[187] Qu L, Yi Z, Shen Y, Lin L, Chen F, Xu Y, Wu Z, Tang H, Zhang X, Tian F, et al. Circular RNA vaccines against SARS-CoV-2 and emerging variants. Cell. 2022;185:1728–1744.e1716. 35460644 10.1016/j.cell.2022.03.044 PMC8971115

